# Contrasting hydrodynamic regimes of submerged pinnacle and emergent coral reefs

**DOI:** 10.1371/journal.pone.0273092

**Published:** 2022-08-16

**Authors:** Gemma F. Galbraith, Benjamin J. Cresswell, Mark I. McCormick, Thomas C. Bridge, Geoffrey P. Jones

**Affiliations:** 1 Marine Biology and Aquaculture, College of Science and Engineering, James Cook University, Townsville, Queensland, Australia; 2 ARC Centre of Excellence for Coral Reef Studies, James Cook University, Townsville, Queensland, Australia; 3 Coastal Marine Field Station, School of Science, University of Waikato, Tauranga, New Zealand; 4 Biodiversity and Geosciences Program, Museum of Tropical Queensland, Queensland Museum Network, Townsville, Queensland, Australia; Secretariat of the Pacific Community, NEW CALEDONIA

## Abstract

Hydrodynamics on coral reefs vary with depth, reef morphology and seascape position. Differences in hydrodynamic regimes strongly influence the structure and function of coral reef ecosystems. Submerged coral reefs on steep-sided, conical bathymetric features like seamounts experience enhanced water circulation as a result of interactions between currents and the abrupt physical structure. There may also be similar interactions between smaller pinnacles and regional water currents in offshore locations (crests > 10 m), while shallow reefs (crests <10 m) may be more subject to surface currents driven by wind, waves and tide. Here we tested whether coral pinnacles experienced stronger and more variable currents compared to emergent reefs at the same depth in both nearshore and offshore positions. Current speeds and temperature were monitored for 12 months at 11 reefs, representing the three different reef categories: submerged offshore pinnacles, emergent offshore reefs and emergent nearshore reefs. We found different patterns in current speeds and temperature among reef types throughout the year and between seasons. Submerged pinnacles exhibited stronger, more variable current speeds compared to both near and offshore emergent reefs. We found seasonal changes in current speeds for pinnacle and nearshore reefs but no variation in current strength on offshore reefs. Whilst instantaneous current directions did reflect the seascape position of individual sites, there was no difference in the directional variability of current speeds between reef types. Annual daily average temperatures at all reef types were not strongly seasonal, changing by less than 2 °C throughout the year. Daily temperature ranges at specific sites however, exhibited considerable variability (annual range of up to 6.5 °C), particularly amongst offshore emergent reefs which experienced the highest temperatures despite greater exposure to regional-scale circulation patterns. Additionally, we found a consistent mismatch between satellite sea surface temperatures and in-situ temperature data, which was on average 2 °C cooler throughout the annual study period. Our results suggest that distinct hydrodynamic processes occur on smaller submerged structures that are physically analogous to seamounts. Our findings highlight important nuances in environmental processes that occur on morphologically distinct coral reef habitats and these are likely to be important drivers for the community dynamics of organisms that inhabit these reefs.

## Introduction

Hydrodynamics are important drivers of ecological communities in all marine habitats [1–3]. ‘Currents’ and waves are water in motion, as driven by forces such as wind and tides. These movements generate strong gradients in physical and biochemical processes that in turn affect ecological processes [4–7]. On shallow emergent coral reefs, wave action driven by stress exerted on the waters surface by wind is a key hydrodynamic influence [8, 9]. Emergent reef crests rise to the upper 10m of water where most wave-driven hydrodynamic energy is focused [10]. Resulting gradients in wave energy, nutrients and temperature regimes drive well-documented patterns in habitat zonation and ecological community structure on coral reefs [11–13]. Submerged reefs, however, have crests at depths below 10–20 m and are consequently less affected by surface waves. This occurs initially because wave energy dissipates with depth but also because the lower resultant energy gradient is then spread over the entire submerged area, not just the crest [14, 15]. Typically found as isolated elevations of the seafloor in mid-shelf or offshore positions, submerged reefs are ubiquitous across global oceans and coastal shelves and constitute significant areas of habitat available for the formation of coral reefs [16–18]. Many aspects of hydrodynamic regimes on shallow emergent coral reefs are relatively well understood [19–21] but we have a limited understanding of the role of hydrodynamics on morphologically distinct submerged coral ecosystems.

Comparative studies between shallow emergent coral reefs and submerged coral reefs have shown considerable variability in ecological communities [15, 22]. Fundamental differences in depth and reef morphology generate distinct biotic and abiotic habitat characteristics at each of these reef types that are important in shaping fish and coral assemblages [15, 23]. Unlike the established pattern of habitat zonation on emergent coral reefs, many submerged coral reefs are found on top of steep sided bathymetric features including banks, shoals and abrupt structures like seamounts, pinnacles, bommies and knolls [24, 25]. Seamounts are large (>1000 m elevation) conical structures typically formed by volcanic activity on oceanic plates [26, 27] and can possess diverse and abundant ecological communities [28–30]. In mid to low latitudes where these structures reach the photic zone (< 300 m) they also provide hard substrate for the formation of coral reefs [31–33]. Pinnacles are superficially similar features, generally smaller than seamounts and usually associated with coastal shelves or as part of other larger submerged topographies [34–37]. In ecological terms, seamounts and pinnacles are physically similar submerged structures that can support diverse and highly abundant ecological communities [30, 38–40].

Interactions between currents and abrupt physical bathymetric features can generate distinct hydrodynamic regimes [41–43]. On seamounts these interactions manifest in localised secondary circulations including fast, turbulent currents, eddies, wakes, internal waves and upwelling [44–50]. Such processes are thought to drive strong biophysical coupling through enhanced mixing and retention of nutrients, leading to high productivity and diverse ecological assemblages [51–54]. In a recent study, we found high abundance, biomass and similar diversity of fishes on a series of small, submerged pinnacles compared to larger emergent reefs in Kimbe Bay, Papua New Guinea [55]. We hypothesise that these patterns may be driven by strong and distinct hydrodynamic regimes. Despite differences in scale and seascape setting, the morphological similarities between pinnacles and seamounts provides a suitable framework for investigating hydrodynamic regimes on pinnacle coral reefs.

**Fig 1:**
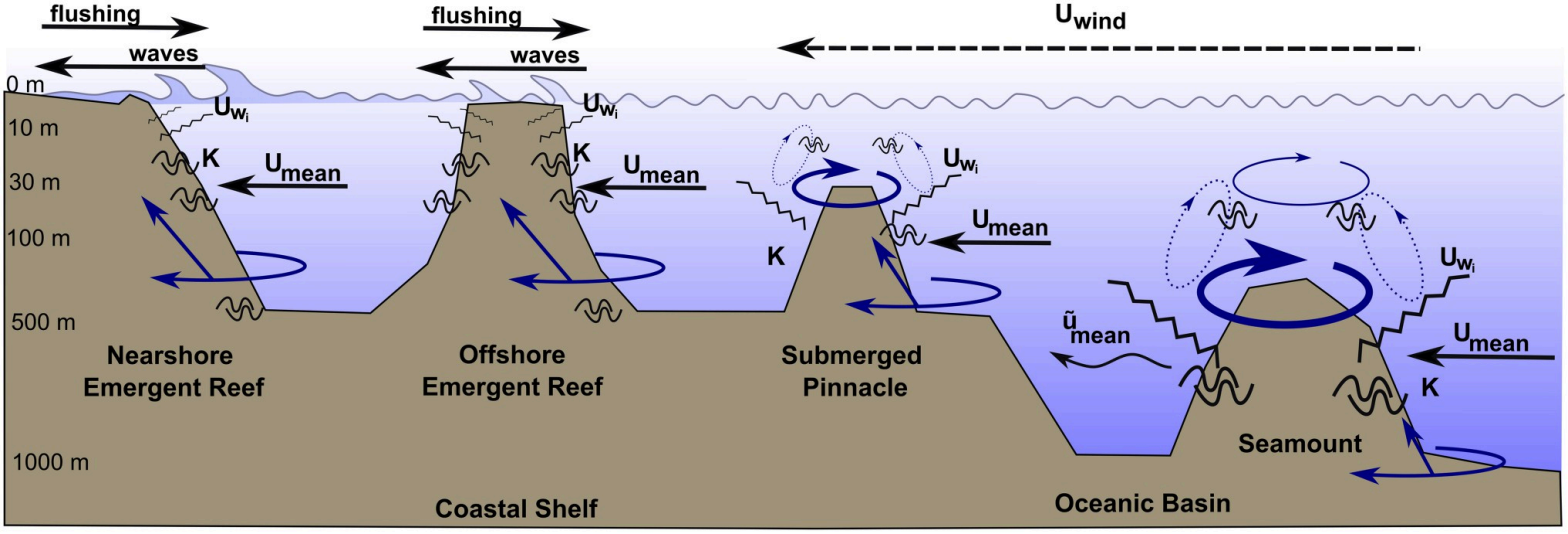
Conceptual diagram of processes occurring on emergent reefs, submerged pinnacles and seamounts. Interactions between abrupt submerged physical structure, incident mean current (U_mean_), eddies (solid blue arrows), internal waves (U_wi_), turbulence (K), oscillating flows and the water column above can lead to doming, upwelling and vertical circulating cells near or above an isolated seamount summit (dashed blue arrows). Adapted from Lavelle and Mohn [43]. Similar processes seem plausible on submerged pinnacles but may not generate larger scale disruptions to current flow like island wakes (${\tilde{u}}_{\text{mean}}$) or trapped cells above the summit (e.g. Taylor Cones). On emergent reefs, most hydrodynamic energy is focused on the crest, and particularly in nearshore positions, is dominated by tides and surface waves. Solid blue arrows represent currents such as eddies moving either above, around or up the slopes of the different bathymetric features. Depth on the y-axis is not to scale and intended only as an aid to distinguish these submerged morphologies.

Hydrodynamic studies on seamounts attribute the formation of accelerated currents and localised eddies to strong interactions between surrounding flows and abrupt conical submerged physical structures [41, 51]. As on seamounts, it seems plausible that currents will be strong and variable on pinnacles at depth, in both speed and direction, due to interactions between prevailing oceanic flow and the abrupt topography. Although emergent coral reefs also represent abrupt physical structures that also have the potential to disrupt large scale oceanographic flows [50, 56, 57], the shallow depth of emergent crests, and therefore lack of significant water column above the crests, reduces the potential for additional hydrodynamic processes. Fig 1 illustrates a conceptualisation of how similar hydrodynamic processes may occur on pinnacles and seamounts, particularly in the water column above the submerged crest. These processes may operate at a smaller scale on submerged pinnacles which, unlike seamounts, are unlikely to disrupt large scale oceanographic flows. However, we hypothesize that the similarly abrupt physical structure could generate localised hydrodynamic responses which subsequently drive variability in ecological patterns and processes on pinnacles. In contrast, shallow emergent crests tend to be dominated by surface wind driven waves and other shallow water hydrodynamics like tidal flushing [5, 20, 58]. Hydrodynamics on submerged pinnacles therefore likely differ to those on emergent reef slopes at the same depth, primarily due to the significant water column above the crest (> 10 m). Further, offshore locations are more exposed to regional currents, weather and oceanic influences so the strength and prevailing direction of currents will also reflect the seascape setting [57, 59, 60]. This may manifest in currents reaching the reef from all directions and could also include exposure to deep circulations and upwellings which tend to be cooler, nutrient rich waters. Whilst submerged pinnacles and emergent reefs exist in both offshore and nearshore seascape positions, it is the depth of crest which will likely determine the nature of water movements influencing the ecological communities found here.

The global degradation of many shallow coral reefs caused by climate change has highlighted the need to understand how future changes in physical environmental conditions will affect ecological processes [61]. This is particularly true for deeper mesophotic coral ecosystems, where environmental conditions differ from shallow emergent reefs, generally down a continuous gradient from 0–300 m [62–66]. Submerged reefs, however, represent a distinct form of deeper reef, with no continuum of habitat extending into shallow water. Many of the proposed localised hydrodynamic responses at seamounts are facilitated by the depth of summit and the extent of the water column above the physical structure [46]. Unlike emergent reefs, submerged coral reefs with deep crests may therefore be subject to additional hydrodynamic processes that may benefit other biophysical dynamics. The lack of comparative studies between emergent and submerged reefs represents a significant knowledge gap in both fundamental ecological processes and also our understanding of how patterns in biodiversity on coral reefs may change.

The aim of this study was to characterise hydrodynamic regimes on a series of submerged pinnacle coral reefs in Kimbe Bay, Papua New Guinea and to compare them with shallow emergent coral reefs in nearshore and offshore seascape positions. We examined current speeds and temperatures for a full year at 11 reefs and assessed how patterns differed between reef morphologies at the same depth. Specifically, we tested hypotheses based on the potential for hydrodynamic similarities between seamounts and pinnacles illustrated in Fig 1:

1. Currents will be stronger and more variable in speed and direction on pinnacle reefs than on emergent reefs at the same depth.
2. Currents are stronger on all offshore reefs (including pinnacles) and nearshore reefs will experience lowest current velocities.
3. Temperature regimes will differ between all reef types as a result of both differences in morphology and seascape position.

## Materials and methods

### Ethics statement

This study was conducted under a Papua New Guinea Research Permit (Research ID 026–18) granted by the National Research Institute, Papua New Guinea. Local permissions to conduct research on the Tamare and Kilu reefs in Kimbe Bay were obtained from Mahonia Na Dari Conservation and Research Centre, Kimbe Bay, West New Britain, Papua New Guinea. All current meters were removed from the reefs after the sampling period was completed.

### Study site and survey design

**Fig 2:**
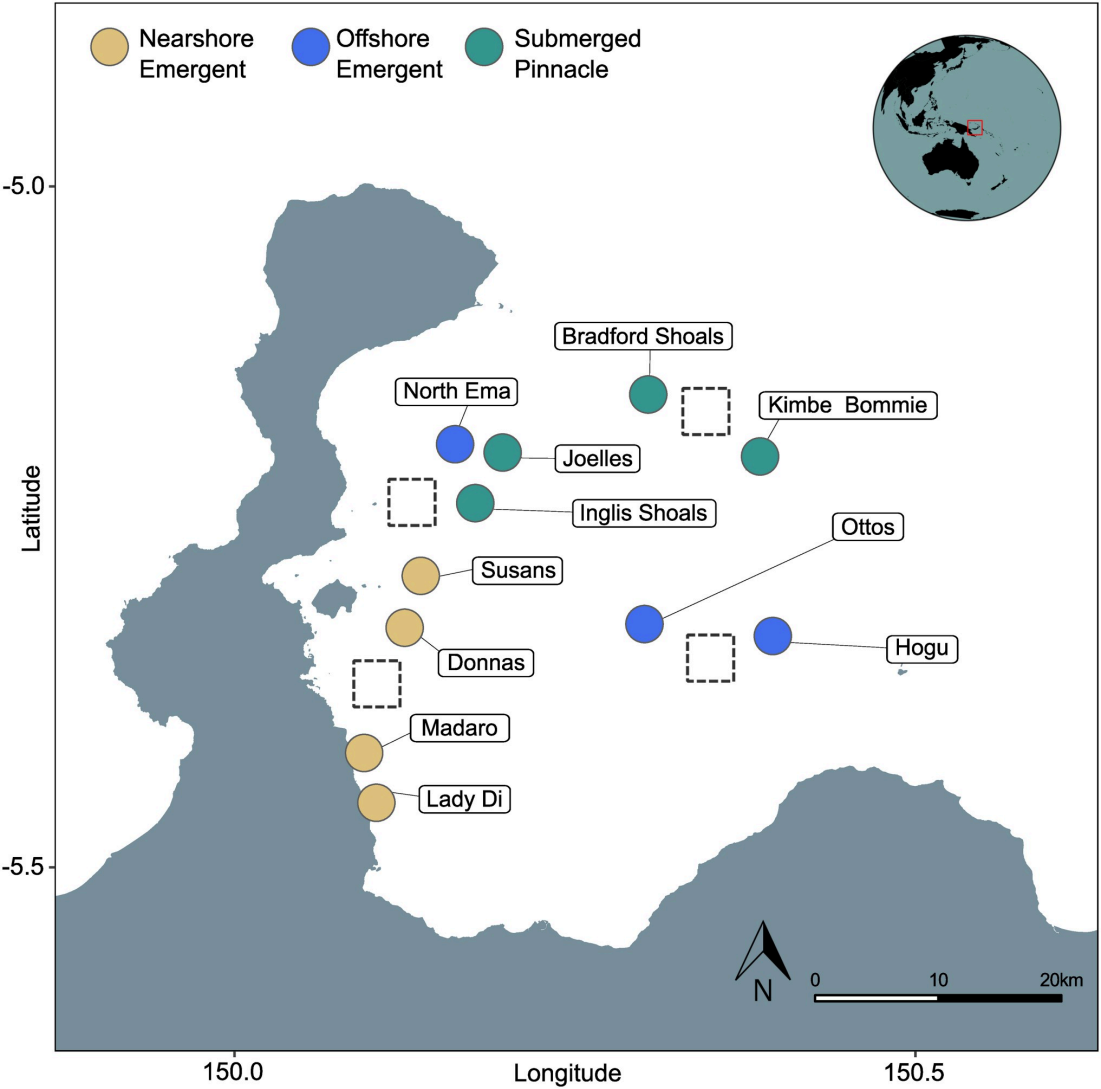
Study site, Kimbe Bay, Papua New Guinea. Reef type for each site is indicated by the colour of marker circle, nearshore emergent (yellow), offshore emergent (blue) and pinnacle (green). Four dashed boxes represent the 4x4 km grids used to collect average Sea Surface Temperatures (SST) over the same annual period.

Kimbe Bay is a relatively large (140 x 70 km) tropical bay to the north of the island of New Britain in the Bismarck Sea (5°30′S, 150°05′E, Fig 2). The bay possesses a diverse seascape including nearshore emergent fringing coral reefs, offshore emergent atolls and guyots as well as numerous submerged coral pinnacles [67], and is therefore an ideal site for a comparative study of differing reef morphologies. The pinnacle summits are small (130–827 m^2^) relatively flat and, being situated in the photic zone with their reef tops between 40–20 m, are capped by coral reef building benthic communities. The sides of both pinnacles and offshore reefs are steep and drop off to 600 m in the bay itself and to over 1000 m on the northern edge of the coastal shelf. Tides in Kimbe Bay are relatively small (1m range at spring tides) and are mostly diurnal [68]. Prevailing seasonal currents and circulation in Kimbe Bay are driven by larger eddies in the eastern Bismarck Sea, which in turn are driven by the southern Equatorial Current [68]. To account for differences in exposure to these regional currents we selected 11 reef sites in both sheltered nearshore and offshore seascape positions of different morphologies; 4 nearshore emergent reefs, 4 offshore emergent reefs and 3 offshore submerged pinnacles. All emergent reefs had crests at depths above 8 m and submerged pinnacle crests were all deeper than 18 m. Nearshore reefs were less than 5 km from mainland and offshore reefs between 9–25 km.

### Data collection

#### In-situ current speed and temperature

To characterise current flow and thermal regimes at each site, Marotte HS Drag-Tilt Current Meter instruments (Marine Geophysics Laboratory, Australia) were installed at 25–30 m depth during May-July 2018. Briefly, these instruments comprise a temperature logger, accelerometer and magnetometer which are calibrated in a controlled environment tank (upright position with no movement) prior to deployment. To account for differences in aspect and zonation as much as possible, current meters were placed on horizontal north-orientated areas of each reef where there were no surrounding obstacles to prevent free movement of the instrument. By standardizing for depth in this way, we ensured that this study examined only sub-surface currents. Consequently, hydrodynamic features characteristic of emergent reefs (e.g. wind waves) are not measured in this study. The instruments recorded temperature (°C), current direction (degree from north) and current speed (m s^-1^) every 10 seconds. Instruments were recovered in September-October 2019 and data downloaded and processed using the software MarotteHSConfig (http://www.marinegeophysics.com.au/software/). This software calculates current speed and direction based on the tilt recorded in comparison to zero-point calibration values. Before statistical analyses, data from each instrument was checked for measurement and potential deployment errors. These quality control checks included temporal consistency (i.e. abnormally high or low values and/or variability in values), consistency in the limits of each measurement (e.g. 0–360° for current direction) and the removal of measurements taken 2 hours before and after deployment (to exclude non-current driven movements and surface temperatures). Additionally, the zero-point calibration values of each instrument were checked pre and post-deployment in a controlled environment and all instruments retained pre-study calibration values. Instruments were painted prior to deployment with a high-strength marine-grade antifoul (International Yacht Paint, Ultra 2). Little to no fouling was observed on any of the instruments.

#### Satellite derived sea surface temperature

Most climate models designed to assess thermal regimes on coral reefs are based on remotely sensed satellite sea surface temperatures (SST). These can differ substantially from temperatures obtained by in-situ loggers and so models seeking to predict the response of reef organisms to ocean warming can lack spatial and temporal accuracy (136). Spatial accuracy includes site-specific depth ranges, therefore, to assess potential differences in satellite derived surface water temperature compared to temperatures at our study depth, we also compared in-situ sub-surface temperatures (at 25–30 m) with sea surface temperatures (SST at <1 m) recorded by remote sensing over the same time period. SSTs were obtained from the 8d MODIS-Aqua satellite time-series, area-averaged 4 μm (day and night) data product using four 4x4 km grids (locations are show by dashed boxes in Fig 2) covering the approximate location of the study reefs via the NASA portal Giovanni v4.34 [69, 70].

### Data analysis

#### Characterising differences in hydrodynamic regimes between reef morphologies

All data were analysed in R 4.0.1. Data from all instruments were collated and standardised to span the time period between September 2018 to September 2019. Kimbe Bay does not exhibit typical seasonal trends in weather and climate [71]. Instead, the area possesses a distinct wet season between December and February and a windy season between June to August. The months in between these “seasons” represent two generally stable transitional periods [71]. We therefore define four seasonal periods in this study; Transitional Period 1 (T1) between September-November, the Wet Season as December-February, Transitional Period 2 (T2) between March-May and the Windy Season as June-August.

Daily values of mean, maximum and minimum current speeds (scalar average, Eq 1) and temperature were calculated for each site. This included a daily average over the full year (annual daily average) and for each season (seasonal daily average). Vector-averaging was used to calculate average current direction for each site (Eqs 2–6), following Grange [72] and implemented in the ‘openair’ package using the ‘timeAverage’ function [73]. Standard deviation vector averaged direction was calculated following Yamartino [74] (Eqs 7 and 8).


$$\bar{u}=\;\frac{1}{n}\;\overset{n}{\underset{i=1}{\sum }}u_{i}$$ (1)

Scalar average current speed ($\overset{-}{u}$) where *u*_*i*_ is the horizontal current speed vector for the *i*-th sample recorded by the current meter (10 second intervals).

Then, for vector averaging of current direction, first directional components are expressed as $\vec{u}$ and $\vec{v}$ which define the direction where the current flow is heading to in radians for each current speed sample (*i*) (Eqs 2 and 3):

$$\vec{u}=(-u_{i})\;sin\left[2\pi \left(\frac{\theta_{i}}{360}\right)\right]$$ (2)

$$\vec{v}=\;(-u_{i})\;cos\left[2\pi \left(\frac{\theta_{i}}{360}\right)\right]$$ (3)

For Eqs 2 and 3, −*u*_*i*_ is the current speed which weights the vectors $\vec{u}$ and $\vec{v}$ by their magnitude and *θ*_*i*_ is the current direction in degrees for each current speed sample in *i*.

To then calculate the resulting vector averaged current direction in degrees ($\overset{-}{\theta }$), both components $\vec{u}$ and $\vec{v}$ are averaged separately to give $\bar{\vec{u}}$ and $\bar{\vec{u}}$ respectively. The addition of the clause (*F*) converts the radians back to degrees, accounting for the appropriate quadrant:

$$\bar{\theta }=\text{arctan}\;\left(\frac{\bar{\vec{u}}}{\bar{\vec{v}}}\right)+F$$ (4)

where

$$F=+\;180\;\text{for}\;\text{arctan}\;\left(\frac{\bar{\vec{u}}}{\bar{\vec{v}}}\right)<\;180$$ (5)

*or*

$$F=-\;180\;\text{for}\;\text{arctan}\;\left(\frac{\bar{\vec{u}}}{\bar{\vec{v}}}\right)>\;180$$ (6)

And for directional standard deviation of averaged unit vectors $\bar{\vec{u}}$ and $\bar{\vec{u}}$:

$$\sigma_{\theta }=\text{arcsin}\;\;\left(\epsilon \right)\;\left[1+\left(\frac{2}{\sqrt{3}}-1\right)\right]\epsilon^{3}$$ (7)

Where *ϵ* is defined as:

$$\epsilon =\sqrt{1-(\bar{\vec{u}}+\bar{\vec{v}}}$$ (8)

#### GLMMs

We used Generalized Linear Mixed effect Models (GLMMs) to compare average measures of temperature and current speed between reef types and between seasons using the package “glmmTMB” [75]. These included comparisons of average daily mean, average daily maximum and average daily minimum. “Site” was included as a random factor in all models to account for between site variability and repeated measures of temperature and currents at each site. Appropriate error distributions were chosen based on standard exploratory techniques and model goodness-of-fit assessed using Q-Q plots (normality), residual plots (homogeneity of variance) in the “DHARMa” package [76]. Generally, temperature models were fitted with a gaussian error structure (identity link) and current speed GLMMs fitted with a gamma error structure (log link). For each GLMM, adjusted Tukey’s tests were used on estimated marginal means (least square) to identify pairwise differences between reef types and seasons using “emmeans” [77]. Estimates and 95% confidence intervals are presented on the response scale, where a confidence interval that does not contain zero suggests evidence for a significant difference between means.

#### Comparing current speed variability

Temporal current speed data characteristically exhibit positively skewed frequency distributions (e.g. Weibull or Gamma). For such distributions, measures of variation based on the mean (e.g. variance and standard deviation) are not robust to extreme outliers or non-normality. Additionally, and in particular for meteorological data, it is the tails of skewed distributions that are most useful when comparing extreme or anomalous values and overall variability [72, 78]. The analysis of variability in wind speed, for example, therefore typically uses the median rather than the mean as a measure of central tendency and deviations from this [79]. We argue that this rational is also applicable to characterising variability in daily average current speeds. To do this, we therefore calculated 25th and 75th percentiles, median, Interquartile Range (IQR) of daily average current speeds at each reef type. The IQR is a measure of the statistical dispersion, higher values of which can indicate the greater possibility of “swings” or ‘pulses” in variables like wind or current speeds [80]. Additionally, we used the Median Absolute Deviation (MAD) as a robust measure of spread ($MAD=Median\;\left({|X}_{i}-\;\tilde{X}|\;\right)$). To compare 25^th^ and 75^th^ percentiles, median and IQR for current speeds amongst reef types we used permutation tests (5000 permutations at 95% confidence level) implemented by the function “pairwisePercentileTest” in the rcompanion package [81].

#### Spatial visualisation of hydrodynamic and thermal spatial patterns

Current rose plots were used to visualise the frequency, strength and direction of currents at each survey reef. Similarly, to visually compare temperatures associated with current speeds recorded at each site, polar plots were also created for each reef. Wind rose and polar plots used hourly means for each reef site during the full 1-year period and were created using the package “openair” [73].

## Results

### Annual and seasonal current regimes—Speed, direction and variability

**Fig 3:**
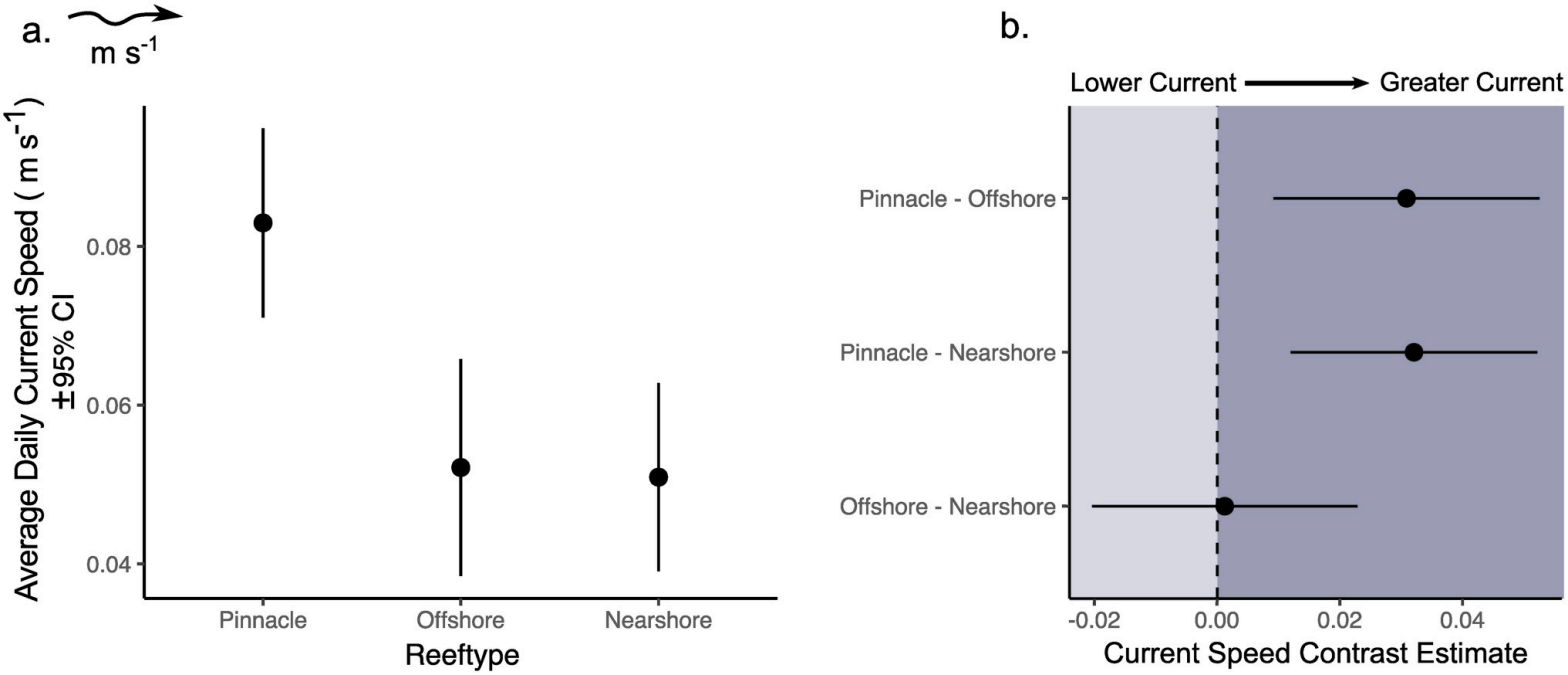
GLMM comparing current speeds between three distinct reef types. **a)** Annual daily mean current speed (m s ^-1^) and 95% confidence intervals at each reef type. **b**) Pairwise contrast estimates based on adjusted Tukey’s tests with 95% confidence intervals. Means are estimated marginal means and contrast estimates represent the difference between each pairwise comparison. 95% confidence intervals for pairwise contrasts are interpreted as significant if the interval does not contain zero.

For the 12 month period between 2018–2019, average daily current speeds were strongest on the pinnacle coral reefs in Kimbe Bay (0.083 m s^-1^; 95% CI = 0.071–0.095), lower on offshore emergent reefs (0.052 m s^-1^; 95% CI = 0.038–0.066) and lowest on nearshore emergent reefs (0.051 m s^-1^; 95% CI = 0.039–0.063). Pairwise contrasts from GLMMs suggest a significant difference in average daily current speeds between pinnacles and both nearshore emergent and offshore emergent reefs respectively, but not between nearshore and offshore reefs which had similar mean daily current speeds (Fig 3). We found no evidence to suggest that annual daily minimum or maximum current speeds differed between reef types. Full results of GLMMs comparing annual mean, maximum and minimum current speed measures are presented in S1 Table in S1 File.

**Fig 4:**
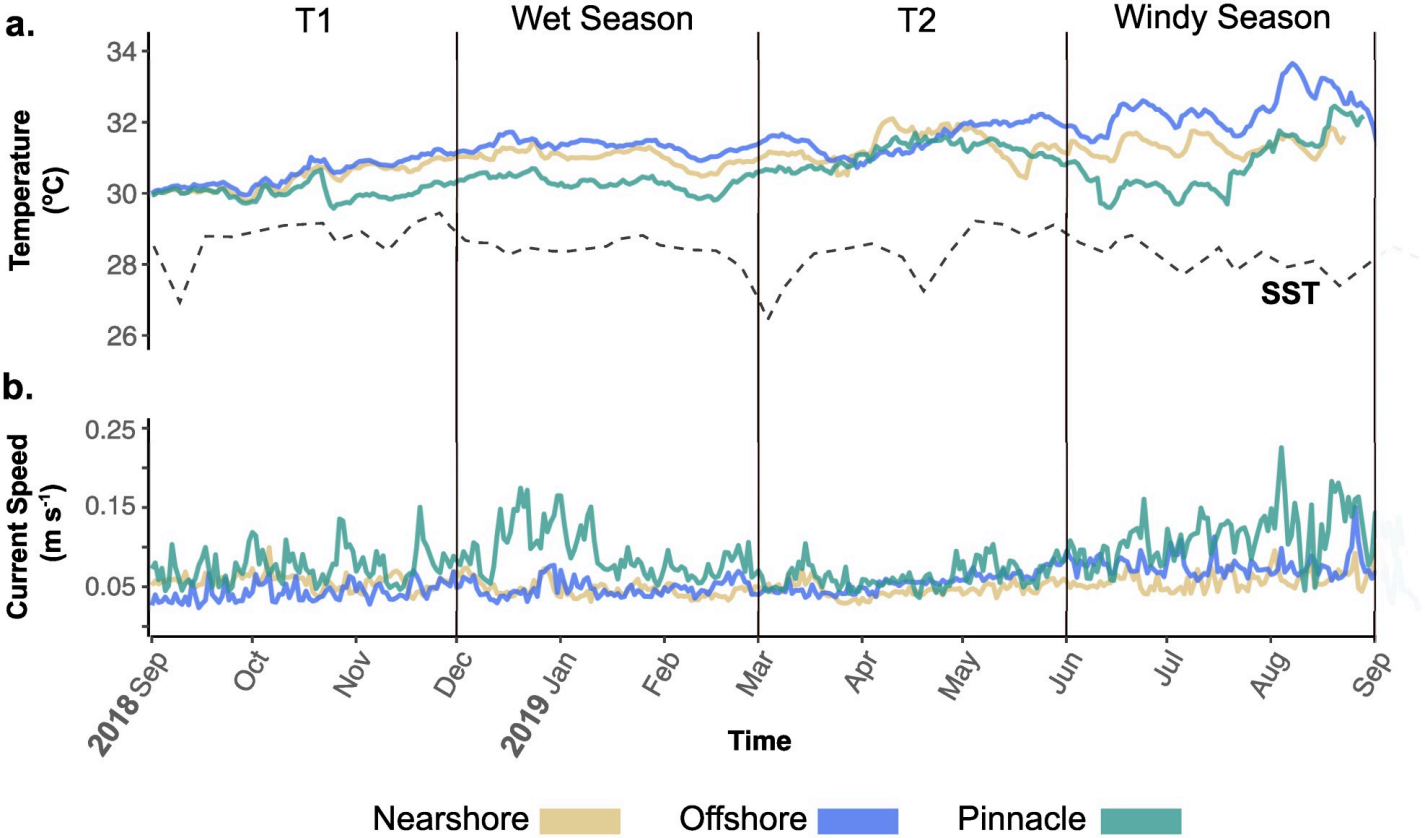
Temperature and current speed time series 2018–2019 at three distinct reef types. **a)** daily mean temperature (°C) and **b)** daily mean current speed (m s ^-1^) at nearshore (yellow, n = 4 sites, 1440 observations), offshore (blue, n = 3 sites, 1080 observations) and pinnacle (green, n = 4 sites, 1440 observations) reefs in Kimbe Bay between Sept 2018 –Sept 2019. The dashed line in the temperature panel (a) represents daily mean sea surface temperature (SST) obtained from remote sensing data and averaged over 4x4km grid squares corresponding with the locations of study reefs in Kimbe Bay (200 daily observations).

**Fig 5:**
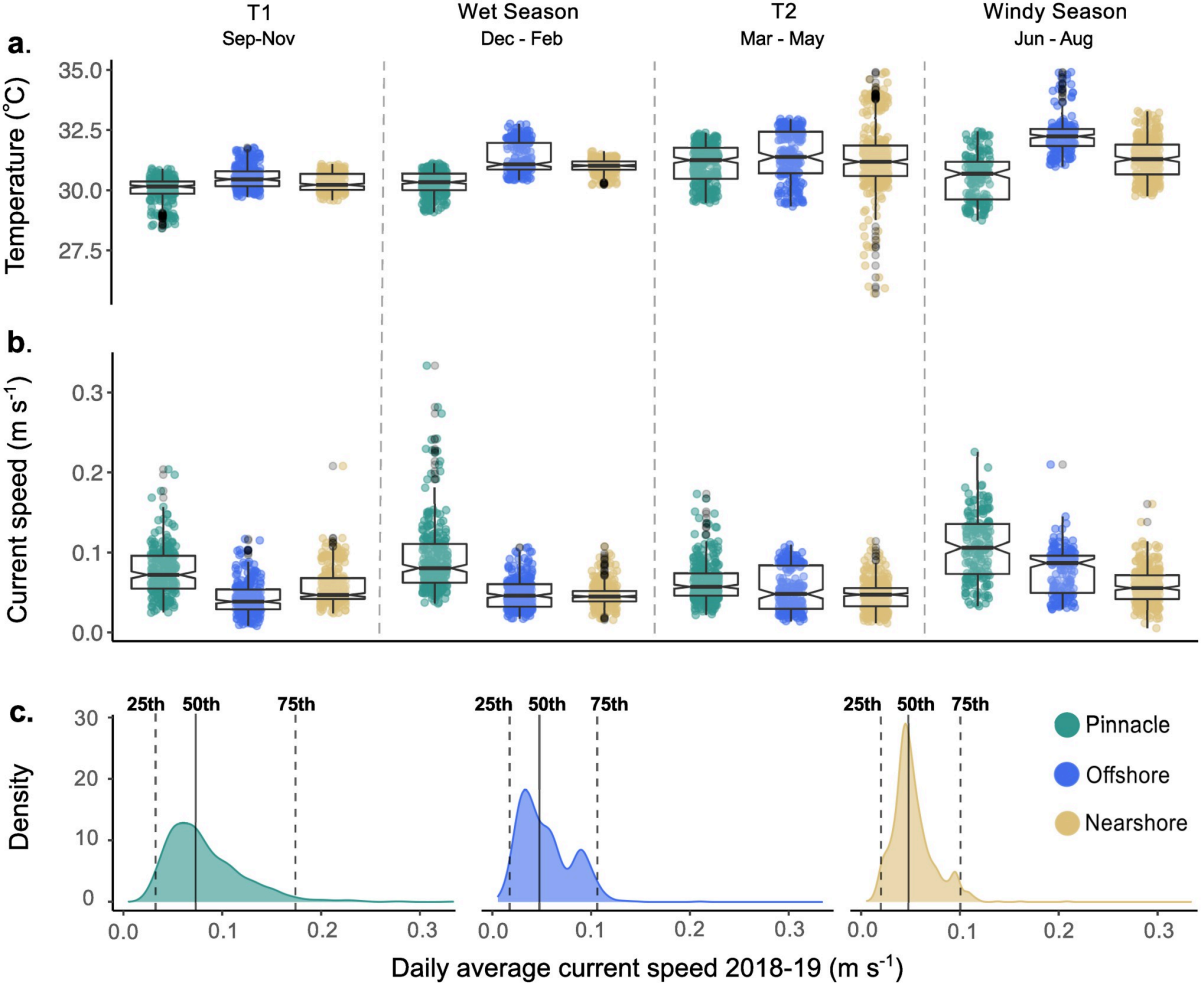
Comparison of temperature and current speed variability between three distinct reef types. Notched boxplots for daily average temperatures **a)** and current speeds **b)** during each seasonal period throughout the year 2018–2019. Boxes represent the interquartile range (25^th^-75^th^ percentiles) and thicker black lines within each box are the median. Notches around the median show 95% confidence intervals where non-overlapping notches suggest evidence of a significant difference between the medians given by m ± 1.58 × IQR/√n. **c)** Frequency density plots for daily average temperatures throughout the year 2018–2019 (all seasons combined). Dashed lines represent the 25^th^ and 75^th^ percentiles. The median is shown as the solid vertical line at the 50^th^ percentile.

Seasonal average daily current speeds on pinnacles were strongest during the wet season (0.10 m s^-1^; 95% CI = 0.09–0.11) and windy season (0.11 m s^-1^; 95% CI = 0.09–0.12) (Figs 4b and 5b). Average current speeds on nearshore reefs exhibited less seasonal variation (range; 0.046–0.058 m s ^-1^) but were strongest during the T1 months (0.057 m s ^-1^; 95% CI = 0.046–0.068) and the windy season (0.056 m s -1; 95% CI = 0.045–0.067). At offshore reefs, there was no evidence to suggest that average daily current speeds differed between any of the seasonal periods (S12 Table in S1 File) ranging between 0.044–0.071 m s^-1^.

Currents on pinnacles exhibited a variable trend throughout the year with repetitive sharp peaks in current speeds in all seasons (Fig 4b). The annual IQR for pinnacle reefs (IQR = 0.048 m s^-1^) was over twice that of nearshore reefs (IQR = 0.021 m s^-1^), and 23% larger than offshore reefs (IQR = 0.039 m s^-1)^ (Fig 5b and 5c, S19 Table in S1 File). Pairwise permutation tests comparing annual current speed IQR were all significant at the 0.05 level (S20 Table in S1 File). This trend was also consistent for median absolute deviation (MAD), which suggests that pinnacles exhibited greatest variability in current speeds throughout the year (median current speed = 0.073 m s^-1^, MAD = 0.034). Nearshore current speed variability was half that of pinnacles (median = 0.048 m s^-1^, MAD = 0.015). Offshore reefs shared the same median current speed as nearshore reefs (0.048 m s ^-1^) but were more variable in strength (MAD = 0.025). See S1 File for all GLMM outputs and pairwise comparisons for annual and seasonal current speed.

**Fig 6:**
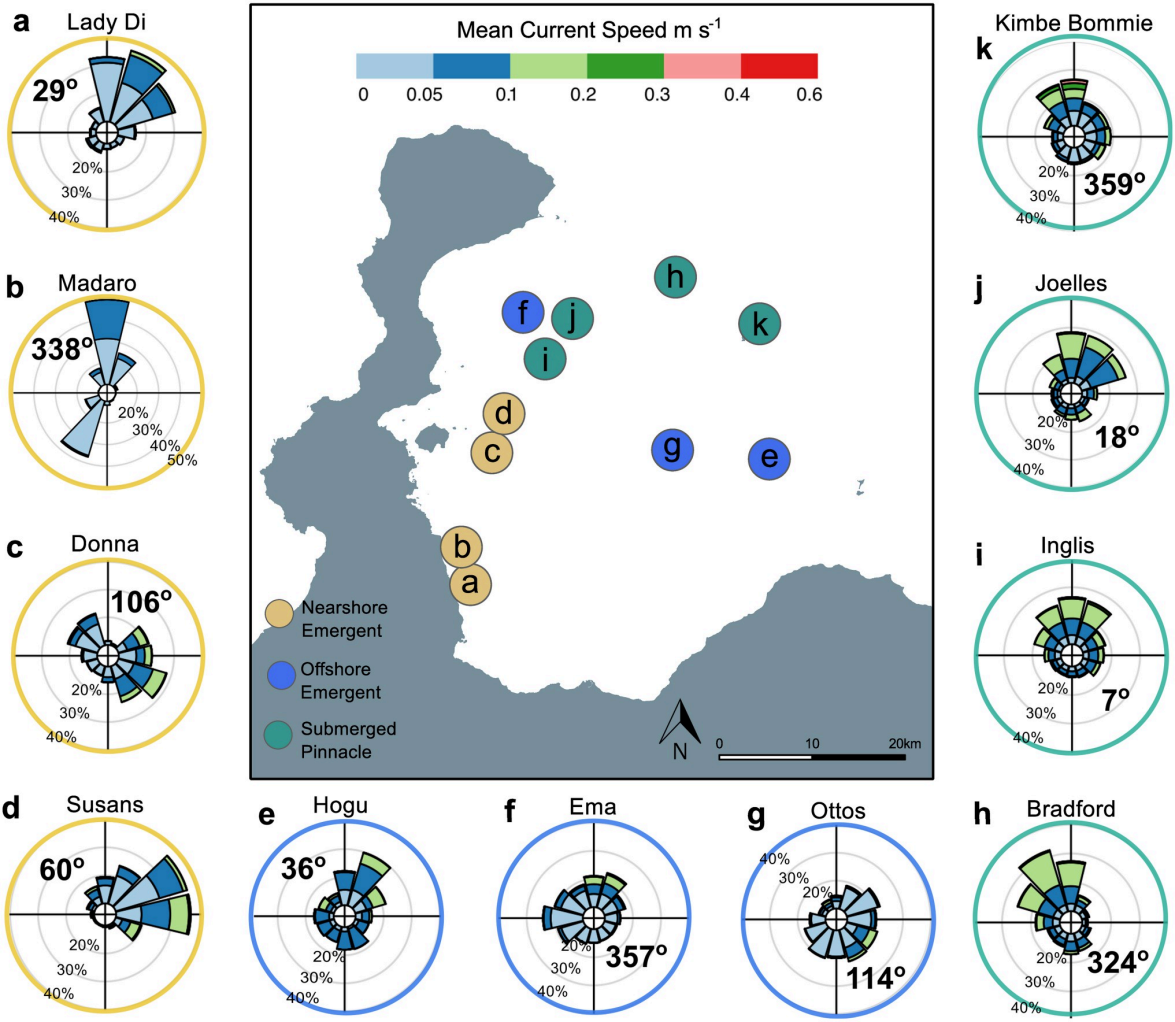
Current rose plots for individual sites. The strength (m s^-1^) and frequency of direction (percentage) of currents is visualised at each site. The bolded degrees in each plot is the average current direction for that reef, calculated by vector analysis. Letters a-k correspond to site locations in Fig 2. Plots were constructed using hourly means from each site for the 1-year study period.

Spatial patterns of the direction frequency of currents and speed varied between reef morphologies and sites (Fig 6). The highest current speeds recorded during the study period occurred on the pinnacle sites (Fig 6h–6k). Current speeds of up to 0.60 m s^-1^ were recorded at the pinnacle site at “Kimbe Bommie”, which was the maximum value observed in this study (S17 Table in S1 File). Although some offshore sites recorded similarly high maximum current speeds (e.g “Otto’s “= 0.59 m s^-1^ and “Ema” = 0.50 m s^-1^, Fig 6g and 6f, S17 Table in S1 File) they did not occur as frequently as on pinnacle sites.

Currents on pinnacles had a dominant north–northwest component (net heading range = 324-18°) and were of magnitudes between 0.1–0.3 m s^-1^ (Fig 6h–6k). On offshore emergent reefs currents were more frequently recorded at less than 0.1 m s^-1^ (Fig 6e–6g) and were generally evenly distributed around 360 degrees. The majority of currents on nearshore reefs did not frequently exceed 0–0.1 m s^-1^ (Fig 6a–6d) but the sites “Donna’s” and “Susan’s” did experience some stronger velocities of up to 0.2 m s^-1^ from the east and south-east. Notably 50% of currents on the nearshore site “Madaro” did not exceed 0.1m s^-1^. Some nearshore sites notably only received currents from certain directions throughout the year. In particular, “Madaro” (Fig 6b) only received northerly or south-westerly components, “Donna” (Fig 6c) mostly south-easterly or north-westerly components and “Susans” (Fig 6d) mostly easterly components. There was however, no statistical evidence to suggest that variability in the direction of prevailing currents differed between reef types, where the random effect of “site” accounted for most variability in the model (GLMM average heading standard deviation S23 Table in S1 File).

### Annual and seasonal temperature regimes

**Fig 7:**
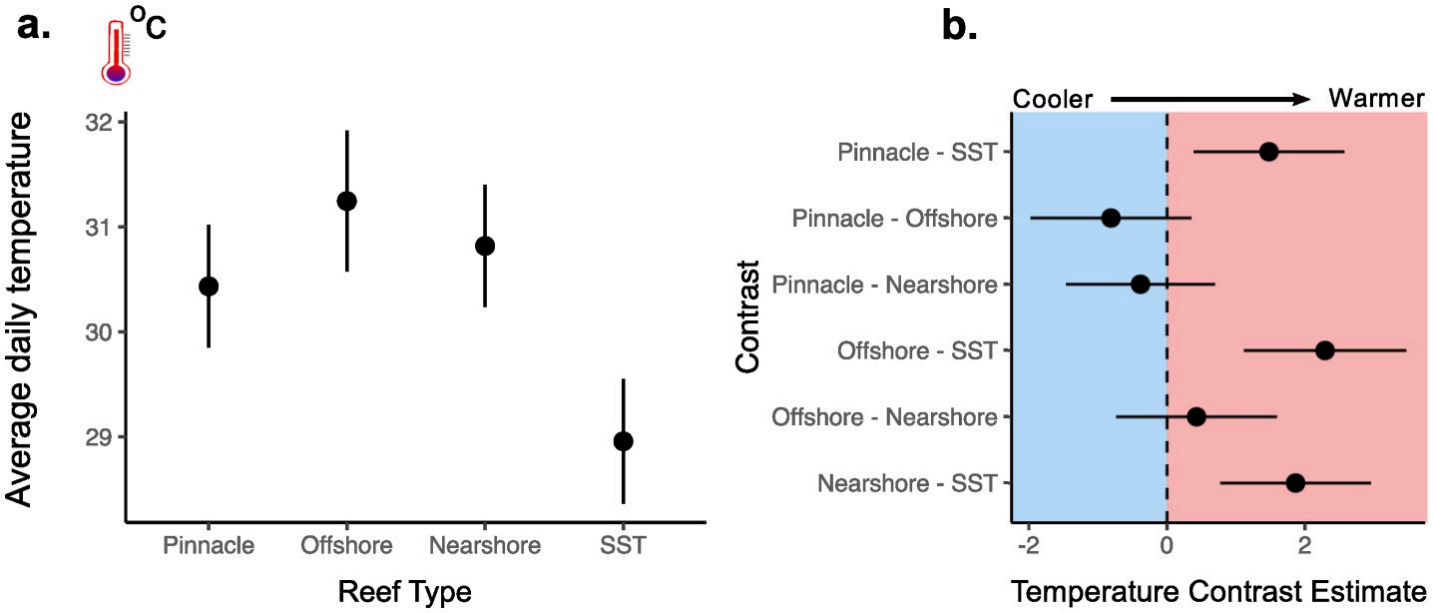
GLMM comparing in-situ temperature between three distinct reef types and sea surface temperature. **a**. Annual daily mean temperature (°C) with 95% confidence intervals at each reef type and for SST. **b**. Pairwise contrast estimates and 95% confidence intervals from adjusted Tukey tests. Means are estimated marginal means and contrast estimates represent the difference between each pairwise contrast. 95% confidence intervals for pairwise contrasts are interpreted as significant if the interval does not cross zero.

During the year-long study period, the highest mean daily sub-surface temperatures were recorded on offshore emergent reefs (31.24 °C; 95% CI = 30.57–31.92) and lowest on pinnacle reefs (30.43 °C; 95% CI = 29.85–31.02). Pairwise contrasts of annual mean daily temperatures amongst reef types were not significantly different but all reef types recorded significantly higher average sub-surface temperatures compared to the average satellite derived SSTs (Fig 7 and S2 Table in S1 File for full GLMM results).

**Fig 8:**
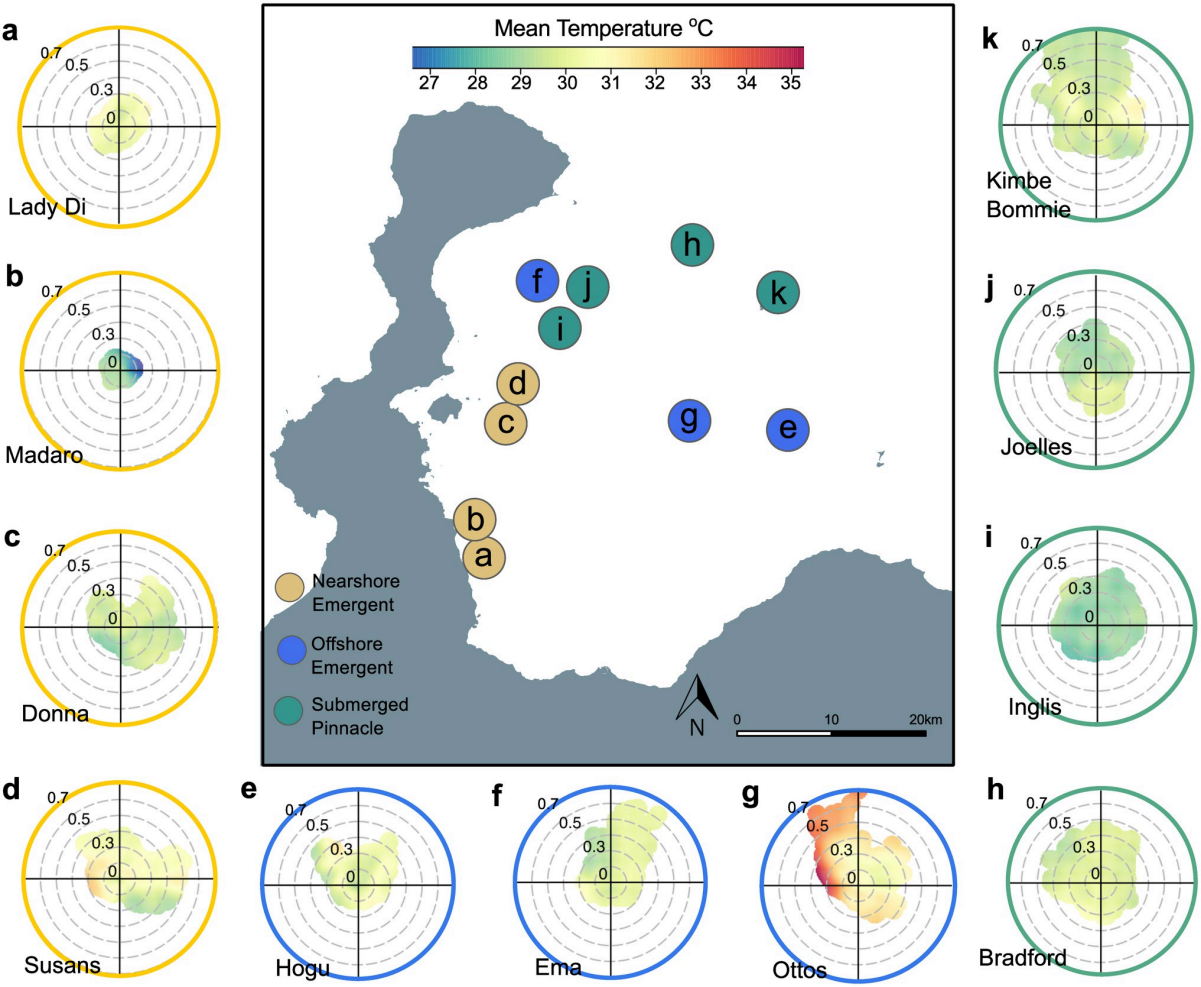
Polar plots for individual sites. The direction (degrees from north), strength (m s ^-1^) and sub-surface temperature (°C at 25–30 m) of currents is shown at each reef site. Letters a-k correspond to site locations in Fig 2. Plots were constructed using hourly means from each site for the 1-year study period, represented by each coloured pixel.

Sub-surface temperature regimes varied between reef types and sites throughout the year (Fig 8). There is some notable variation among sites within each of the three reef categories. One nearshore site (Fig 8b; Madaro) was particularly cool and recorded a minimum temperature of 28.75°C during the survey period. The offshore reef “Ottos” recorded the highest daily average temperature of 35.24 °C (Fig 8g) whereas other offshore sites “Ema” and “Hogu” were cooler (Fig 8e and 8f). All pinnacle sites generally displayed cooler temperatures (Fig 8h–8k) which did not exceed 30.62 °C as an average annual maximum (S2 Table in S1 File).

Within each reef type group, we found evidence to suggest some thermal variation in daily mean temperatures between seasons (S14-S16 Tables in S1 File and Fig 5). The magnitude of these changes, however, was small (<2 °C). The warmest temperatures recorded at each reef type did not occur within the same seasons. On pinnacles, warmest daily mean water temperatures were recorded in the T2 period (31.12 °C; 95% CI = 30.51–31.72) whereas offshore reefs and nearshore reefs were warmest in the windy season (offshore 32.19 °C; 95% CI = 31.53–32.86 and nearshore 31.26 °C; 95% CI = 30.4–32.03). All reefs were coolest during September—November (T1). Between reef types, the only significant differences were found in the wet season, where pinnacle reefs were 1.03 °C cooler than offshore reefs (contrast estimate CI = -1.92–-0.135—see S14-S16 Tables in S1 File for all seasonal temperature GLMMs and pairwise comparisons).

Sea surface temperature (SST) measured by satellite was consistently lower than temperatures recorded by in-situ loggers at all reef types. The annual trend broadly followed temperature patterns on the study reefs but with some inverse peaks and, was on average lower by 2.05 °C during the year (Fig 4). Full GLMM outputs and pairwise estimates for temperature comparisons between reef types and sea surface temperature are reported in S2-S6 Tables in S1 File.

## Discussion

### Distinct hydrodynamics on submerged coral pinnacles are defined by strong and variable current speeds

Our results were consistent with the expectation that pinnacles and offshore reefs would experience the strongest currents, likely as a result of greater exposure to prevailing regional currents and weather. Cross-shelf gradients in exposure and environmental stressors are well documented on coral reefs that span continental and coastal shelves [21, 82, 83]. Reefs in nearshore, mid-shelf and offshore positions therefore experience distinct environmental regimes which lead to variation in habitat structure and ecological assemblages [84–87]. Although the offshore reefs in Kimbe Bay experienced a similar absolute range of current velocities as pinnacles (Table 1), these high current speeds were not sustained, as reflected by lower mean and median daily values. As such, we found no evidence to suggest that average daily current speeds were different between nearshore and offshore reefs (Fig 3, S1 Table in S1 File). Additionally, the IQR of current speeds on pinnacles was not only larger than near and offshore reefs, but was also skewed toward higher, more extreme values. This suggests that despite seascape position, hydrodynamics at the depths surveyed were more similar on all emergent reefs and that pinnacles consistently experienced stronger and more variable current velocities.

**Table 1:** Reef type summaries of temperature and current speeds. Absolute minimum, maximum and range of temperature and current speeds recorded at each reef type during the study period. Site-specific values are listed in S17 Table in S1 File.

	Temperature (°C)			Current Speed (m s ^-1^)		
Reef Type	Min	Max	Range	Min	Max	Range
Pinnacle	28.14	32.84	4.70	0.0000	0.6030	0.6030
Offshore	28.75	35.24	6.50	0.0004	0.5976	0.5975
Nearshore	25.49	35.25	9.76	0.0004	0.4132	0.4131

We propose that these differences are a product of distinct interactions between submerged physical structures and impinging oceanic flows that can form in the water column above the submerged crest (> 10 m depth). While these phenomena are not of the scale associated with seamounts, such as Taylor cones [88] or enclosed circulation cells [89], our results suggest that there may be localised interactions generating diverted or funnelled currents on submerged coral pinnacles. Some primary factors that determine the character of flow at seamounts include seamount height, local ocean density stratification (dependent on salinity, temperature and pressure), seamount morphology (base width and slope) and impinging steady and oscillatory currents [43]. The pinnacles in our study rise from depths of 600m in the middle of the bay. The bay also possesses several distinct internal circulations that are also influenced by stronger regional gyres (Steinberg et al. 2006), and the coastal shelf of West New Britain descends steeply to depths of over 1000m in the north of the bay. Currents reaching pinnacles could therefore likely be modified by the same factors that determine dynamic currents on seamounts, albeit on a smaller scale. Although sub-surface currents clearly reach the deep slopes of all reef structures, refraction and the propagation of internal waves can distribute energy horizontally around the structure or vertically up the slope [90–92]. In this way, much hydrodynamic energy continues to be focused on the shallow crest of emergent reefs or is dissipated around the structure. Refraction and propagation almost certainly occur in this way on submerged features, however the water column above the summit facilitates additional localized hydrodynamic responses which may then be retained and focused on the crest at greater depths (Fig 1). Flow modification is often more pronounced on elevated features of a seamount [93, 94] and our study supports others which show that these effects also occur on isolated pinnacles and other bathymetric highs [39]. Although we controlled for variability in exposure and aspect in the placement of current meters (choosing north facing, horizontal areas free from obstruction) a more detailed study utilising multiple current meters in different reef zones would allow further examination of fine-scale sub-surface water movement patterns at or around certain features of each reef.

These processes have important biological implications as stronger currents tend to enhance feeding opportunities for some organisms and can therefore shape ecological assemblages [93, 95, 96]. Additionally, increased turbulence can re-suspend sediment and detritus that has been trapped on the summit, generating further benefits to sessile suspension feeders like sponges and corals [41]. Temporary eddies and small vortexes can also enhance the availability of dissolved particulate matter and nutrients in multiple directions by downflux, upwelling flux and retention over a summit [43, 97]. In offshore settings with clearer waters, this enhanced nutrient availability may increase complex coral cover at greater depths. Further, phototrophic taxa on the summits of submerged geomorphologies receive greater intensity and duration of daylight and, lower sediment loads due to lack of shading and shedding from reef habitat above [42, 98, 99]. For fishes, although peaks in diversity can vary with depth on emergent reefs [100–102] enhanced hydrodynamic environments coupled with complex habitat could explain observations of high species richness and abundance of fishes at small, isolated submerged coral reefs, despite greater absolute depth.

Although net heading directions could be qualitatively characterised for each reef type, we did not find any statistical differences in the variability of current direction. Each reef likely experiences some degree of unique current flow as a product of individual seascape position [68]. The lack of currents from certain directions on nearshore reefs, for example, seems to reflect coastal, more sheltered locations within the bay. Despite the relatively high sensitivity of the instruments used to record current speed in this study, our measurements are limited to horizontal water movement only. Therefore, whilst our findings do present evidence for distinct hydrodynamics on submerged pinnacle coral reefs, this preliminary study did not capture the full range of hydrodynamic processes likely occurring at each site. Not least because oceanographic modelling of fine-scale and often transient processes like upwellings and localised eddies requires a measure of vertical flow. Such high-resolution oceanography data, including vertical and horizontal components, is typical in modelling structural flow dynamics and physical properties (density layers, mixing etc) of the water column surrounding seamounts [103–105].

Whilst variability in current speed, direction and water temperature can be used to infer the presence of more complex current patterns, the use of advanced instrumentation (e.g. acoustic doppler current profilers and conductivity, temperature, depth sensors) would greatly benefit future research building on the current study.

### Lack of annual temperature variability but high spatial variability

The three reef types investigated in this study exhibited stable daily average thermal conditions during 2018–2019, varying by 2 °C or less. Although the global average seasonal range for tropical coral reefs is also limited to ~4–5 °C, [106] our results suggest remarkably weak seasonality of annual temperature regimes in Kimbe Bay during the 1 year study period (2018–2019).

Historically thermally stable environments, like low latitude coral reefs, allow organisms to specialize within narrow temperature ranges [107–111] and individuals can maximise the efficiency of multiple life-history processes [112–114]. For example, the coral reef sea urchin, *Diadema setosum*, exhibits longer breeding seasons at lower latitudes which can be near continuous in equatorial locations [115, 116]. Srinivasan and Jones [71] also found a similar lack of water temperature seasonality in Kimbe Bay and, additionally, that extended breeding and recruitment periods for two families of coral reef fishes (Labridae and Pomacentridae) were more linked with changes in the monsoonal climate (rainfall and wind speed) than temperature. For corals, although seasonal temperature changes are a key predictor of peak mass spawning [117], reproductive synchrony is also evident in equatorial regions where temperature changes are markedly reduced [118, 119]. The lack of annual thermal variability in Kimbe Bay therefore likely contributes to favourable year-round environmental conditions for a wide range of coral reef taxa.

The predicted rise in global sea surface temperature (2.0–4.8°C) by the end of the century [120, 121] however, renders coral reef species particularly vulnerable to climate change, as individuals are already living close to their thermal maxima [122, 123]. Heat stress, arising from both rising global average temperature and the increasing frequency and severity of extreme heating events, significantly reduces key processes in growth, reproduction, metabolic performance and ecological interactions [124–129]. This can scale to ecosystem-wide habitat degradation, global coral bleaching and population mass mortality [130–135]. The exceptionally low seasonal change in water temperatures found in our study therefore also suggests that ecological communities here may be even more sensitive to thermal stress events than other low latitude locations. Indeed, long-term declines in coral cover in Kimbe Bay have been reported since 1996 with parallel declines in fish biodiversity [136].

It is increasingly recognised that the response of marine organisms and ecosystems to rising SST will not only be determined by the capacity of individuals to adapt to thermal stress in the long term (years to decades) but also to fluctuations over much shorter periods (e.g. diurnal to weekly patterns) [137, 138]. Whilst our study found low variability in annual mean temperatures between reef types, some individual reefs exhibited markedly high fluctuations in daily temperatures. The nearshore reef “Donnas” and offshore reef “Ottos” had the largest absolute maximum and minimum ranges temperatures throughout the year (6.5 °C and 6.3 °C respectively). For nearshore reefs this could reflect the proximity of these sites to higher inputs of cooler fresh water from run-off and river plumes [139] alternating with warmer temperatures due to rapid surface heating of shallow nearshore waters. Stronger and warmer currents at some offshore sites could be due to the location of these sites within pockets of enclosed circulations that have less exchange with cooler seasonal gyres in the wider Bismarck Sea [68]. Although the underlying mechanism driving these spatial differences in temperature variability cannot be determined from this study, our results highlight that ecological responses to rising and more variable global temperatures will likely not be uniform for all coral reefs, even at the scale of a small tropical bay seascape.

### Mismatched in-situ temperatures and SST

Interestingly, our in-situ temperature data was between 1.63–2.43 °C warmer than the average annual temperature obtained from satellite-derived SST data (28.90 °C) (Fig 7). This discrepancy could be partly explained by the larger spatial window over which satellite-derived temperature data are calculated, however, it also supports the wide recognition of mismatches between global models of sea surface temperature and empirical data collected in the field [140]. Cooler surface waters could be explained by the semi-enclosed nature of the bay together with its large river catchments and high annual rainfall (3,180 mm per annum [68]). This has important consequences for the mixing of water layers and therefore differences in energy and nutrient cycles between depths [141]. Lower absolute temperature ranges on pinnacles could also represent some degree of isothermal doming of cooler deeper water over the summit, which is another prominent hydrodynamic process on seamounts [142, 143]. The potential inverse stratification reported here is consistent with our empirical observations while diving in Kimbe Bay, where there is typically a noticeably colder layer of water between the surface and ~5 m (measured by dive computers). Additionally, the average temperature over the same period as this study recorded by a shallow in-situ logger (29.97 °C at 2 m) [144] was also lower than all in-situ temperatures at each reef type (30.43–31.24 °C). However, these in-situ surface temperature data are currently limited to one site and depth and therefore lack sufficiently replicated observations for robust statistical comparisons. Clearly a more detailed investigation of water column properties from the surface to greater depths would be required to comprehensively describe and confirm stratification patterns in Kimbe Bay. This would need to include accurate in-situ measurements from the surface across the depth gradient using more advanced instrumentation (e.g. a CTD)

### Potential influence of regional seasonality and hydrodynamics driving mismatches in thermal maxima

Kimbe Bay is located in the Western Pacific Warm Pool (WPWP), one of the warmest parts of the ocean [145]. The WPWP is also strongly connected to El Niño Southern Oscillation (ENSO) events [146]. The onset of an ENSO event in December 2018, which extended until August 2019, may reflect the upward trend in temperatures at all reef types throughout the year. Kimbe Bay does not experiences typical seasonality but instead two distinct seasons associated with the Southeast Asian/Australian monsoon system [68, 71]. Between November to March (wet season) the northwest monsoon system brings higher rainfall and prevailing easterly currents. In May to October (windy season) a drier season prevails with strong south-easterly trade winds which can reverse the direction of local currents and gyres in the Bismarck Sea [68]. These changes clearly influence temperature regimes in Kimbe Bay throughout the year; however, the seasonal differences we observed were not consistent between reef types. The transitional season between March and May (T2) was the warmest season on pinnacles, but between June and August (windy season) for nearshore and offshore reefs. One explanation for this could be variability in hydrodynamics. The higher T2 temperatures on pinnacles, for example, corresponded with the lowest and least variable current speeds at these sites. Temperatures then dropped in the windy season which corresponded with increased frequency of strong spikes in current velocities. Although currents were also highest in the windy season for nearshore and offshore reefs, this did not correspond with lower temperatures. Nearshore and offshore reefs did experience some spikes in current speed throughout the year but these patterns were not of the same magnitude. Further analysis is warranted to directly assess the relationship between current speed and water temperature in this study, particularly the nature and extent of upwellings which have not been investigated in Kimbe Bay.

Understanding physical processes in tropical marine environments is of increasing importance, particularly processes that could confer resilience by thermal buffering. Hydrodynamics like upwelling, internal waves and high flow rates can moderate the ecological impacts of thermal stress and on some reefs, may offer temporary or seasonal refuge [147–151]. Although hydrodynamics have a strong influence on thermodynamics on all reefs [152–155], differences in current regimes between emergent and submerged morphologies may lead to divergent responses to thermal stress in some taxa [156]. Temperatures are generally lower on deeper reefs [157] but the extent to which site-specific currents influence thermal regimes is not well known. Depth does not represent a panacea for coral reef communities [62, 158–160], but thermal refuges may be more pronounced on submerged bathymetries with strong hydrodynamic environments. Pinnacles had the smallest absolute range in temperature throughout the year (4.7 °C) suggesting more stable thermal environments with restricted maximum temperatures. One of the most pronounced differences we found was during the windy season when the average temperature on submerged pinnacle reefs was 1.75 °C cooler than on offshore emergent reef sites. If these differences are generated by unique submerged structure-flow interactions, it highlights the importance of reef morphology rather than depth alone for the future distribution of coral reef communities under climate change.

## Conclusions

While hydrodynamic forcing and thermodynamics on fringing, barrier and atoll reef systems have been well investigated [21, 154], few studies have examined differences in these processes between submerged and emergent reefs in one geographic region. Our results highlight that different reef morphologies can experience distinct spatio-temporal variation in hydrodynamics at the same depth over the same annual period. Hydrodynamics on coral reefs are important ecological drivers and our results provide a good foundation for further studies examining patterns of biodiversity on submerged reefs. Future work should also include the collection of high-resolution oceanographic data (e.g. salinity, chlorophyll, dissolved oxygen) to enhance our understanding of biophysical coupling on submerged coral reefs and significantly expand on the preliminary results presented in this study. Our results likely extend to other submerged pinnacle coral reefs but studies in additional locations are required to determine the generality of these patterns. Hydrodynamics that enhance productivity and drive thermoregulatory mechanisms are especially important in light of future warming from climate change and global degradation of shallow reefs. Although depth and isolation are often suggested to afford some degree of refuge, distinct environmental conditions generated by abrupt submerged morphologies highlights the importance of understanding the physical environments of all coral reefs in future coral seascapes.

## Supporting information

S1 File Full GLMM results and supplementary tables. (DOCX)

S2 File Inclusivity in global research. (DOCX)
