# Supplementary material for: Contrasting hydrodynamic regimes of submerged pinnacle and emergent coral reefs
Source: PLoS One. 2022 Aug 16;17(8):e0273092. doi: 10.1371/journal.pone.0273092 (PMC9380949; doi:10.1371/journal.pone.0273092)
Supplement: S1 File — (DOCX) [file pone.0273092.s001.docx]

**Table S1** – GLMM (Gamma -Log Link) results comparing annual mean daily current speed (m s^-1^) between reef types for the deployment period Sept 18 – Sept 19. Pairwise contrasts and 95% confidence intervals are presented based on adjusted Tukeys method. Pinnacles n = 1440 daily mean values, Offshore n = 1080, Nearshore n = 1440

| *Reef Type* | *estimate* | | *SE* | *lower.CL* | *upper.CL* | *contrast* | *estimate* | *SE* | *lower.CL* | *upper.CL* |
| --- | --- | --- | --- | --- | --- | --- | --- | --- | --- | --- |
| **Annual Mean Current** | | | |  |  |  | | |  |  |
| Pinnacle | 0.083 | 0.006 | | 0.071 | 0.095 | Pinnacle - Offshore | 0.031 | 0.009 | 0.009 | 0.053 |
| Offshore | 0.052 | 0.007 | | 0.038 | 0.066 | Pinnacle - Nearshore | 0.032 | 0.009 | 0.012 | 0.052 |
| Nearshore | 0.051 | 0.006 | | 0.039 | 0.063 | Offshore - Nearshore | 0.001 | 0.009 | -0.020 | 0.023 |
| **Average Annual Max Current** | | | | | | | | | | |
| Pinnacle | 0.17 | 0.02 | | 0.13 | 0.21 | Pinnacle - Offshore | 0.02 | 0.03 | -0.04 | 0.09 |
| Offshore | 0.15 | 0.02 | | 0.11 | 0.19 | Pinnacle - Nearshore | 0.05 | 0.03 | -0.01 | 0.11 |
| Nearshore | 0.12 | 0.02 | | 0.09 | 0.16 | Offshore - Nearshore | 0.02 | 0.03 | -0.04 | 0.09 |
| **Average Annual Min Current** | | | | |  |  |  |  |  |  |
| Pinnacle | 0.018 | 0.006 | | 0.008 | 0.029 | Pinnacle - Offshore | 0.005 | 0.008 | -0.015 | 0.024 |
| Offshore | 0.014 | 0.006 | | 0.001 | 0.026 | Pinnacle - Nearshore | -0.001 | 0.008 | -0.019 | 0.018 |
| Nearshore | 0.019 | 0.006 | | 0.008 | 0.030 | Offshore - Nearshore | -0.005 | 0.008 | -0.025 | 0.014 |

| *Reef Type* | *estimate* | | *SE* | *lower.CL* | *upper.CL* | *contrast* | *estimate* | *SE* | *lower.CL* | *upper.CL* |
| --- | --- | --- | --- | --- | --- | --- | --- | --- | --- | --- |
| **Annual Mean Temperature** | | | | | |  | | |  |  |
| Pinnacle | 30.43 | 0.30 | | 29.85 | 31.02 | Pinnacle – Offshore | -0.81 | 0.45 | -1.98 | 0.35 |
| Offshore | 31.24 | 0.34 | | 30.57 | 31.92 | Pinnacle – Nearshore | -0.39 | 0.42 | -1.47 | 0.70 |
| Nearshore | 30.82 | 0.30 | | 30.23 | 31.40 | Pinnacle – SST | 1.48 | 0.43 | 0.38 | 2.57 |
| SST | 28.96 | 0.30 | | 28.36 | 29.55 | Offshore – Nearshore | 0.43 | 0.45 | -0.74 | 1.59 |
|  |  |  | |  |  | Offshore – SST | 2.29 | 0.46 | 1.11 | 3.47 |
|  |  |  | |  |  | Nearshore – SST | 1.86 | 0.42 | 0.77 | 2.95 |
| **Average Annual Max Temperature** | | | | |  |  |  |  |  |  |
| Pinnacle | 30.62 | 0.35 | | 29.94 | 31.30 | Pinnacle – Offshore | -0.80 | 0.53 | -2.04 | 0.44 |
| Offshore | 31.42 | 0.40 | | 30.63 | 32.20 | Pinnacle – Nearshore | -0.36 | 0.49 | -1.51 | 0.79 |
| Nearshore | 30.98 | 0.35 | | 30.30 | 31.66 | Offshore – Nearshore | 0.44 | 0.53 | -0.80 | 1.68 |
| **Average Annual Min Temperature** | | | | |  |  |  |  |  |  |
| Pinnacle | 30.16 | 0.29 | | 29.58 | 30.74 | Pinnacle – Offshore | -0.84 | 0.45 | -1.89 | 0.21 |
| Offshore | 31.00 | 0.34 | | 30.33 | 31.66 | Pinnacle – Nearshore | -0.42 | 0.42 | -1.39 | 0.56 |
| Nearshore | 30.58 | 0.29 | | 30.00 | 31.15 | Offshore – Nearshore | 0.42 | 0.45 | -0.63 | 1.47 |

**Table S2** – GLMM (Gaussian -Identity Link) results comparing annual mean daily temperature (^o^C) between reef types and SST for the deployment period Sept 18 – Sept 19. Site was included as a random factor within the model structure. Pairwise contrasts and 95% confidence intervals are presented based on adjusted Tukeys method. Pinnacles n = 1440 daily mean values, Offshore n = 1080, Nearshore n = 1440 and SST values n = 200

**Table S3** – GLMM (Gaussian -Identity Link) results comparing mean daily temperature (^o^C) between reef types in Transition Period 1 (T1 is Sep- Nov 2018). Pairwise contrasts and 95% confidence intervals are presented based on adjusted Tukeys method. Pinnacles n = 360 daily mean values, Offshore n = 276, Nearshore n = 360

| *Reef Type* | *estimate* | | *SE* | *lower.CL* | *upper.CL* | *contrast* | *estimate* | *SE* | *lower.CL* | *upper.CL* |
| --- | --- | --- | --- | --- | --- | --- | --- | --- | --- | --- |
| **T1 Mean Temperature** | | | |  |  |  | | |  |  |
| Pinnacle | 29.93 | 0.20 | | 29.54 | 30.32 | Pinnacle - Offshore | -0.61 | 0.31 | -1.40 | 0.17 |
| Offshore | 30.54 | 0.23 | | 30.09 | 30.99 | Pinnacle - Nearshore | -0.37 | 0.28 | -1.1 | 0.356 |
| Nearshore | 30.30 | 0.20 | | 29.91 | 30.69 | Pinnacle - SST | 0.78 | 0.29 | 0.04 | 1.52 |
| SST | 29.15 | 0.21 | | 28.74 | 29.55 | Offshore - Nearshore | 0.24 | 0.30 | -0.54 | 1.03 |
|  |  |  | |  |  | Offshore - SST | 1.39 | 0.31 | 0.60 | 2.19 |
|  |  |  | |  |  | Nearshore - SST | 1.15 | 0.29 | 0.41 | 1.89 |
| **Average T1 Max Temp** | | | |  |  |  |  |  |  |  |
| Pinnacle | 30.08 | 0.23 | | 29.63 | 30.52 | Pinnacle - Offshore | -0.61 | 0.35 | -1.43 | 0.21 |
| Offshore | 30.68 | 0.26 | | 30.17 | 31.20 | Pinnacle - Nearshore | -0.35 | 0.32 | -1.10 | 0.41 |
| Nearshore | 30.42 | 0.23 | | 29.98 | 30.87 | Offshore - Nearshore | 0.26 | 0.35 | -0.56 | 1.08 |
| **Average T1 Min Temp** | | | | |  |  |  |  |  |  |
| Pinnacle | 29.67 | 0.23 | | 29.22 | 30.12 | Pinnacle - Offshore | -0.64 | 0.35 | -1.46 | 0.18 |
| Offshore | 30.31 | 0.26 | | 29.79 | 30.82 | Pinnacle - Nearshore | -0.41 | 0.32 | -1.17 | 0.35 |
| Nearshore | 30.08 | 0.23 | | 29.63 | 30.53 | Offshore - Nearshore | 0.23 | 0.35 | -0.60 | 1.05 |

**Table S4** – GLMM (Gaussian -Identity Link) results comparing mean daily current speed (m s^-1^) between reef types in the Wet seasons (Dec 2018 – Feb 2019). Pairwise contrasts and 95% confidence intervals are presented based on adjusted Tukeys method. Pinnacles n = 360 daily mean values, Offshore n = 276, Nearshore n = 360

| *Reef Type* | *estimate* | | *SE* | *lower.CL* | *upper.CL* | *contrast* | *estimate* | *SE* | | *lower.CL* | | *upper.CL* | |
| --- | --- | --- | --- | --- | --- | --- | --- | --- | --- | --- | --- | --- | --- |
| **Wet Season Mean Temperature** | | | | | |  | | |  | |  | |  |
| Pinnacle | 30.30 | 0.25 | | 29.81 | 30.77 | Pinnacle - Offshore | -1.03 | 0.35 | | -1.92 | | -0.14 | |
| Offshore | 31.32 | 0.25 | | 30.84 | 31.80 | Pinnacle - Nearshore | -0.71 | 0.35 | | -1.6 | | 0.18 | |
| Nearshore | 31.01 | 0.25 | | 30.53 | 31.49 | Pinnacle - SST | 1.22 | 0.33 | | 0.38 | | 2.06 | |
| SST | 29.071 | 0.22 | | 28.64 | 29.50 | Offshore - Nearshore | 0.31 | 0.35 | | -0.58 | | 1.20 | |
|  |  |  | |  |  | Offshore - SST | 2.25 | 0.33 | | 1.41 | | 3.09 | |
|  |  |  | |  |  | Nearshore - SST | 1.94 | 0.33 | | 1.09 | | 2.78 | |
| **Average Wet Season Max Temp** | | | | |  |  |  |  | |  | |  | |
| Pinnacle | 30.47 | 0.30 | | 29.89 | 31.06 | Pinnacle - Offshore | -1.02 | 0.42 | | -2.01 | | -0.03 | |
| Offshore | 31.49 | 0.30 | | 30.91 | 32.08 | Pinnacle - Nearshore | -0.70 | 0.42 | | -1.69 | | 0.29 | |
| Nearshore | 31.17 | 0.30 | | 30.58 | 31.75 | Offshore - Nearshore | 0.32 | 0.42 | | -0.67 | | 1.31 | |
| **Average Wet Season Min Temp** | | | | |  |  |  |  | |  | |  | |
| Pinnacle | 30.02 | 0.30 | | 29.44 | 30.61 | Pinnacle - Offshore | -1.06 | 0.42 | | -2.06 | | -0.07 | |
| Offshore | 31.08 | 0.30 | | 30.50 | 31.67 | Pinnacle - Nearshore | -0.75 | 0.42 | | -1.74 | | 0.24 | |
| Nearshore | 30.77 | 0.30 | | 30.19 | 31.36 | Offshore - Nearshore | 0.31 | 0.42 | | -0.68 | | 1.30 | |

**Table S5** – GLMM (Gaussian -Identity Link) results mean daily temperature (^o^C) between reef types in Transition season 2 (T2, Mar – May 2019). Pairwise contrasts and 95% confidence intervals are presented based on adjusted Tukeys method. Pinnacles n = 368 daily mean values, Offshore n = 276, Nearshore n = 368

| *Reef Type* | *estimate* | | *SE* | *lower.CL* | *upper.CL* | *contrast* | *estimate* | *SE* | *lower.CL* | *upper.CL* |
| --- | --- | --- | --- | --- | --- | --- | --- | --- | --- | --- |
| **T2 Mean Temperature** | | | |  |  |  | | |  |  |
| Pinnacle | 31.12 | 0.47 | | 30.19 | 32.05 | Pinnacle - Offshore | -0.30 | 0.72 | -2.16 | 1.56 |
| Offshore | 31.42 | 0.55 | | 30.35 | 32.49 | Pinnacle - Nearshore | -0.12 | 0.67 | -1.84 | 1.61 |
| Nearshore | 31.24 | 0.47 | | 30.31 | 32.16 | Pinnacle - SST | 2.35 | 0.68 | 0.59 | 4.11 |
| SST | 28.77 | 0.49 | | 27.81 | 29.74 | Offshore - Nearshore | 0.19 | 0.72 | -1.67 | 2.04 |
|  |  |  | |  |  | Offshore - SST | 2.65 | 0.73 | 0.76 | 4.54 |
|  |  |  | |  |  | Nearshore - SST | 2.46 | 0.68 | 0.71 | 4.22 |
| **Average T2 Max Temp** | | | |  |  |  |  |  |  |  |
| Pinnacle | 31.39 | 0.54 | | 30.33 | 32.45 | Pinnacle - Offshore | -0.23 | 0.83 | -2.17 | 1.71 |
| Offshore | 31.62 | 0.62 | | 30.40 | 32.84 | Pinnacle - Nearshore | -0.05 | 0.76 | -1.85 | 1.74 |
| Nearshore | 31.44 | 0.54 | | 30.38 | 32.50 | Offshore - Nearshore | 0.18 | 0.82 | -1.76 | 2.12 |
| **Average T2 Min Temp - Gamma Inverse link** | | | | |  |  |  |  |  |  |
| Pinnacle | 30.79 | 0.54 | | 29.73 | 31.84 | Pinnacle - Offshore | -0.37 | 0.83 | -2.32 | 1.58 |
| Offshore | 31.15 | 0.63 | | 29.91 | 32.40 | Pinnacle - Nearshore | -0.13 | 0.76 | -1.92 | 1.66 |
| Nearshore | 30.91 | 0.54 | | 29.85 | 31.97 | Offshore - Nearshore | 0.24 | 0.83 | -1.72 | 2.19 |

**Table S6** – GLMM (Gaussian -Identity Link) results comparing mean daily temperature (^o^C) between reef types in the Windy Season (June – Aug 2019). Pairwise contrasts and 95% confidence intervals are presented based on adjusted Tukeys method. Pinnacles n = 368 daily mean values, Offshore n = 276, Nearshore n = 368

| *Reef Type* | *estimate* | | *SE* | *lower.CL* | *upper.CL* | *contrast* | *estimate* | *SE* | *lower.CL* | | *upper.CL* | |  |
| --- | --- | --- | --- | --- | --- | --- | --- | --- | --- | --- | --- | --- | --- |
| **Windy Mean Temperature** | | | | | | | | | |  | |  | |
| Pinnacle | 30.82 | 0.49 | | 29.86 | 31.77 | Pinnacle – Offshore | -1.48 | 0.77 | -3.46 | | 0.51 | |  |
| Offshore | 32.29 | 0.60 | | 31.12 | 33.46 | Pinnacle – Nearshore | -0.24 | 0.65 | -1.90 | | 1.43 | |  |
| Nearshore | 31.05 | 0.42 | | 30.22 | 31.88 | Pinnacle – SST | 2.01 | 0.65 | 0.34 | | 3.69 | |  |
| SST | 28.80 | 0.43 | | 27.96 | 29.65 | Offshore – Nearshore | 1.24 | 0.73 | -0.64 | | 3.12 | |  |
|  |  |  | |  |  | Offshore – SST | 3.49 | 0.73 | 1.60 | | 5.38 | |  |
|  |  |  | |  |  | Nearshore – SST | 2.25 | 0.60 | 0.70 | | 3.80 | |  |
| **Average Windy Max Temp – Gamma inverse link** | | | | | |  |  |  |  | |  | |  |
| Pinnacle | 30.96 | 0.60 | | 29.79 | 32.14 | Pinnacle – Offshore | -1.52 | 1.00 | -3.88 | | 0.84 | |  |
| Offshore | 32.48 | 0.81 | | 30.90 | 34.07 | Pinnacle – Nearshore | -0.22 | 0.80 | -2.09 | | 1.66 | |  |
| Nearshore | 31.18 | 0.53 | | 30.15 | 32.22 | Offshore – Nearshore | 1.30 | 0.96 | -0.96 | | 3.56 | |  |
| **Average Windy Min Temp** | | | | |  |  |  |  |  | |  | |  |
| Pinnacle | 30.52 | 0.57 | | 29.41 | 31.64 | Pinnacle – Offshore | -1.48 | 0.95 | -3.72 | | 0.76 | |  |
| Offshore | 32.00 | 0.76 | | 30.50 | 33.50 | Pinnacle – Nearshore | -0.29 | 0.76 | -2.07 | | 1.50 | |  |
| Nearshore | 30.81 | 0.50 | | 29.82 | 31.80 | Offshore – Nearshore | 1.19 | 0.91 | -0.96 | | 3.34 | |  |

**Table S7** – GLMM (Gamma – log link) results comparing mean daily current speed (m s ^-1^) between reef types in Transition Season 1 (T1, Sept – Nov 2018). Pairwise contrasts and 95% confidence intervals are presented based on adjusted Tukeys method. Pinnacles n = 360 daily mean values, Offshore n = 276, Nearshore n = 360

| *Reef Type* | *estimate* | | *SE* | *lower.CL* | *upper.CL* | *contrast* | *estimate* | *SE* | *lower.CL* | | *upper.CL* |
| --- | --- | --- | --- | --- | --- | --- | --- | --- | --- | --- | --- |
| **T1 Mean Current Speed** | | | | | | | | | |  |  |
| Pinnacle | 0.079 | 0.006 | | 0.066 | 0.091 | Pinnacle - Offshore | 0.035 | 0.009 | 0.013 | | 0.058 |
| Offshore | 0.043 | 0.007 | | 0.029 | 0.057 | Pinnacle - Nearshore | 0.022 | 0.009 | 0.002 | | 0.043 |
| Nearshore | 0.056 | 0.006 | | 0.044 | 0.068 | Offshore - Nearshore | -0.013 | 0.009 | -0.035 | | 0.009 |
| **Average T1 Max Current Speed** | | | | | | |  |  |  | |  |
| Pinnacle | 0.167 | 0.021 | | 0.126 | 0.209 | Pinnacle - Offshore | 0.026 | 0.032 | -0.050 | | 0.102 |
| Offshore | 0.141 | 0.024 | | 0.094 | 0.189 | Pinnacle - Nearshore | 0.038 | 0.030 | -0.033 | | 0.108 |
| Nearshore | 0.130 | 0.021 | | 0.089 | 0.171 | Offshore - Nearshore | 0.011 | 0.032 | -0.064 | | 0.087 |
| **Average T1 Min Current Speed** | | | | | | |  |  |  | |  |
| Pinnacle | 0.014 | 0.006 | | 0.002 | 0.026 | Pinnacle - Offshore | 0.009 | 0.009 | -0.012 | | 0.030 |
| Offshore | 0.005 | 0.007 | | -0.008 | 0.018 | Pinnacle - Nearshore | -0.011 | 0.008 | -0.030 | | 0.009 |
| Nearshore | 0.025 | 0.006 | | 0.013 | 0.036 | Offshore - Nearshore | -0.019 | 0.009 | -0.040 | | 0.001 |

**Table S8** – GLMM (Gamma – log link) results comparing mean daily current speed (m s ^-1^) between reef types in the Wet Season (Dec 18 – Feb 19). Pairwise contrasts and 95% confidence intervals are presented based on adjusted Tukeys method. Pinnacles n = 360 daily mean values, Offshore n = 276, Nearshore n = 360

| *Ref Type* | *estimate* | | *SE* | *lower.CL* | *upper.CL* | *contrast* | *estimate* | *SE* | *lower.CL* | | *upper.CL* |
| --- | --- | --- | --- | --- | --- | --- | --- | --- | --- | --- | --- |
| **Wet Mean Current Speed – Gamma log link** | | | | | |  | | | |  |  |
| Pinnacle | 0.092 | 0.010 | | 0.072 | 0.112 | Pinnacle - Offshore | 0.045 | 0.011 | 0.018 | | 0.072 |
| Offshore | 0.048 | 0.005 | | 0.037 | 0.058 | Pinnacle - Nearshore | 0.046 | 0.011 | 0.019 | | 0.072 |
| Nearshore | 0.047 | 0.005 | | 0.037 | 0.057 | Offshore - Nearshore | 0.001 | 0.007 | -0.016 | | 0.018 |
| **Average Wet Max Current Speed** | | | | | | |  |  |  | |  |
| Pinnacle | 0.177 | 0.034 | | 0.110 | 0.244 | Pinnacle - Offshore | 0.038 | 0.040 | -0.057 | | 0.132 |
| Offshore | 0.139 | 0.021 | | 0.097 | 0.181 | Pinnacle - Nearshore | 0.042 | 0.040 | -0.051 | | 0.135 |
| Nearshore | 0.135 | 0.020 | | 0.096 | 0.174 | Offshore - Nearshore | 0.004 | 0.029 | -0.064 | | 0.073 |
| **Average Wet Min Current Speed** | | | | | | |  |  |  | |  |
| Pinnacle | 0.019 | 0.005 | | 0.010 | 0.028 | Pinnacle - Offshore | 0.010 | 0.007 | -0.005 | | 0.026 |
| Offshore | 0.009 | 0.005 | | -0.000 | 0.018 | Pinnacle - Nearshore | 0.006 | 0.007 | -0.009 | | 0.022 |
| Nearshore | 0.013 | 0.005 | | 0.004 | 0.022 | Offshore - Nearshore | -0.004 | 0.007 | -0.019 | | 0.012 |

**Table S9** – GLMM (Gamma – log link) results comparing mean daily current speed (m s ^-1^) between reef types in Transition Season 2 (T2, March – May 2019). Pairwise contrasts and 95% confidence intervals are presented based on adjusted Tukeys method. Pinnacles n = 368 daily mean values, Offshore n = 276, Nearshore n = 368

| *Reef Type* | *estimate* | | *SE* | *lower.CL* | *upper.CL* | *contrast* | *estimate* | *SE* | *lower.CL* | | *upper.CL* |
| --- | --- | --- | --- | --- | --- | --- | --- | --- | --- | --- | --- |
| **T2 Mean Current Speed** | | | |  |  |  | | | |  |  |
| Pinnacle | 0.065 | 0.008 | | 0.048 | 0.081 | Pinnacle - Offshore | 0.014 | 0.013 | -0.016 | | 0.045 |
| Offshore | 0.051 | 0.010 | | 0.032 | 0.070 | Pinnacle - Nearshore | 0.018 | 0.012 | -0.010 | | 0.046 |
| Nearshore | 0.046 | 0.008 | | 0.030 | 0.063 | Offshore - Nearshore | 0.004 | 0.013 | -0.026 | | 0.034 |
| **T2 Mean Max Current Speed** | | | | |  |  |  |  |  | |  |
| Pinnacle | 0.151 | 0.017 | | 0.118 | 0.184 | Pinnacle - Offshore | 0.019 | 0.025 | -0.040 | | 0.079 |
| Offshore | 0.131 | 0.019 | | 0.094 | 0.169 | Pinnacle - Nearshore | 0.035 | 0.024 | -0.020 | | 0.090 |
| Nearshore | 0.116 | 0.017 | | 0.083 | 0.148 | Offshore - Nearshore | 0.016 | 0.025 | -0.044 | | 0.075 |
| **T2 Mean Min Current Speed** | | | | |  |  |  |  |  | |  |
| Pinnacle | 0.009 | 0.007 | | -0.005 | 0.024 | Pinnacle - Offshore | -0.007 | 0.011 | -0.033 | | 0.018 |
| Offshore | 0.017 | 0.008 | | 0.000 | 0.033 | Pinnacle - Nearshore | -0.009 | 0.010 | -0.033 | | 0.015 |
| Nearshore | 0.018 | 0.007 | | 0.004 | 0.033 | Offshore - Nearshore | -0.002 | 0.011 | -0.028 | | 0.024 |

**Table S10** – GLMM (Gamma – log link) results comparing mean daily current speed (m s ^-1^) between reef types in the Windy Season (June-Aug 2019). Pairwise contrasts and 95% confidence intervals are presented based on adjusted Tukeys method. Pinnacles n = 368 daily mean values, Offshore n = 276, Nearshore n = 368

| *Reef Type* | *estimate* | | *SE* | *lower.CL* | *upper.CL* | *contrast* | *estimate* | *SE* | *lower.CL* | *upper.CL* |
| --- | --- | --- | --- | --- | --- | --- | --- | --- | --- | --- |
| **Windy Mean Current Speed** | | | |  |  |  | | |  |  |
| Pinnacle | 0.096 | 0.014 | | 0.068 | 0.123 | Pinnacle - Offshore | 0.018 | 0.022 | -0.033 | 0.070 |
| Offshore | 0.077 | 0.017 | | 0.044 | 0.111 | Pinnacle - Nearshore | 0.041 | 0.019 | -0.002 | 0.085 |
| Nearshore | 0.055 | 0.012 | | 0.031 | 0.078 | Offshore - Nearshore | 0.023 | 0.021 | -0.026 | 0.072 |
| **Average Windy Max Current Speed** | | | | |  |  |  |  |  |  |
| Pinnacle | 0.194 | 0.022 | | 0.150 | 0.238 | Pinnacle - Offshore | 0.018 | 0.035 | -0.063 | 0.100 |
| Offshore | 0.176 | 0.027 | | 0.123 | 0.228 | Pinnacle - Nearshore | 0.059 | 0.029 | -0.010 | 0.128 |
| Nearshore | 0.135 | 0.019 | | 0.097 | 0.173 | Offshore - Nearshore | 0.040 | 0.033 | -0.037 | 0.118 |
| **Average Windy Min Current Speed** | | | | |  |  |  |  |  |  |
| Pinnacle | 0.030 | 0.012 | | 0.007 | 0.053 | Pinnacle - Offshore | -0.005 | 0.018 | -0.048 | 0.039 |
| Offshore | 0.035 | 0.014 | | 0.007 | 0.062 | Pinnacle - Nearshore | 0.016 | 0.015 | -0.021 | 0.052 |
| Nearshore | 0.014 | 0.010 | | -0.006 | 0.034 | Offshore - Nearshore | 0.020 | 0.017 | -0.021 | 0.061 |

**Table S11** – GLMM (Gamma – log link) results comparing mean daily current speed (m s ^-1^) between seasons on four pinnacle reefs in Kimbe Bay. Pairwise contrasts and 95% confidence intervals are presented based on adjusted Tukeys method. T1 n = 360, Wet Season n = 360, T2 n = 368, Windy Season n = 368

| *Season* | *estimate* | | *SE* | *lower.CL* | *upper.CL* | *contrast* | | *estimate* | *SE* | *lower.CL* | | *upper.CL* |
| --- | --- | --- | --- | --- | --- | --- | --- | --- | --- | --- | --- | --- |
| **Mean Current Speed** | | | | | |  | | | | |  |  |
| T1 | 0.08 | 0.00 | | 0.07 | 0.08 | T1 - Wet | | -0.02 | 0.00 | -0.03 | | -0.01 |
| Wet | 0.10 | 0.01 | | 0.09 | 0.11 | T1 - T2 | | 0.01 | 0.00 | 0.01 | | 0.02 |
| T2 | 0.06 | 0.00 | | 0.06 | 0.07 | T1 - Windy | | -0.03 | 0.01 | -0.05 | | -0.02 |
| Windy | 0.11 | 0.01 | | 0.09 | 0.12 | Wet - T2 | | 0.03 | 0.00 | 0.02 | | 0.04 |
|  |  |  | |  |  | Wet - Windy | | -0.01 | 0.00 | -0.02 | | -0.00 |
|  |  |  | |  |  | T2 - Windy | | -0.04 | 0.01 | -0.06 | | -0.03 |
| **Average Max Current Speed** | | | | | | |  | |  |  | |  |
| T1 | 0.16 | 0.01 | | 0.14 | 0.18 | T1 - Wet | | -0.02 | 0.01 | -0.04 | | -0.00 |
| Wet | 0.18 | 0.01 | | 0.15 | 0.21 | T1 - T2 | | 0.01 | 0.01 | -0.00 | | 0.03 |
| T2 | 0.15 | 0.01 | | 0.13 | 0.17 | T1 - Windy | | -0.05 | 0.01 | -0.08 | | -0.02 |
| Windy | 0.21 | 0.02 | | 0.17 | 0.24 | Wet - T2 | | 0.03 | 0.01 | 0.02 | | 0.05 |
|  |  |  | |  |  | Wet - Windy | | -0.03 | 0.01 | -0.05 | | -0.01 |
|  |  |  | |  |  | T2 - Windy | | -0.06 | 0.01 | -0.09 | | -0.03 |
| **Average Min Current Speed** | | | | |  |  | |  |  |  | |  |
| T1 | 0.02 | 0.00 | | 0.01 | 0.02 | T1 - Wet | | -0.00 | 0.00 | -0.01 | | 0.00 |
| Wet | 0.02 | 0.00 | | 0.01 | 0.03 | T1 - T2 | | 0.00 | 0.00 | -0.00 | | 0.01 |
| T2 | 0.01 | 0.00 | | 0.00 | 0.02 | T1 - Windy | | -0.02 | 0.00 | -0.03 | | -0.01 |
| Windy | 0.04 | 0.00 | | 0.03 | 0.04 | Wet - T2 | | 0.01 | 0.00 | 0.00 | | 0.01 |
|  |  |  | |  |  | Wet - Windy | | -0.02 | 0.00 | -0.02 | | -0.01 |
|  |  |  | |  |  | T2 - Windy | | -0.03 | 0.00 | -0.03 | | -0.02 |
| **Table S12** – GLMM (Gamma – log link) results comparing mean daily current speed (m s ^-1^) between seasons on three offshore reefs in Kimbe Bay. Pairwise contrasts and 95% confidence intervals are presented based on adjusted Tukeys method. T1 n = 276, Wet Season n = 276, T2 n = 276, Windy Season n = 276 | | | | | | | | | | | | |
| *Season* | *estimate* | *SE* | | *lower.CL* | *upper.CL* | *contrast* | | *estimate* | *SE* | *lower.CL* | | *upper.CL* |
| **Mean Current Speed** | | | | | |  | | | | |  |  |
| T1 | 0.04 | 0.01 | | 0.02 | 0.06 | T1 - Wet | | -0.01 | 0.00 | -0.01 | | -0.00 |
| Wet | 0.05 | 0.01 | | 0.03 | 0.07 | T1 - T2 | | -0.01 | 0.00 | -0.01 | | -0.00 |
| T2 | 0.05 | 0.01 | | 0.03 | 0.07 | T1 - Windy | | -0.03 | 0.00 | -0.04 | | -0.03 |
| Windy | 0.07 | 0.01 | | 0.06 | 0.09 | Wet - T2 | | -0.00 | 0.00 | -0.01 | | 0.00 |
|  |  |  | |  |  | Wet - Windy | | -0.03 | 0.00 | -0.03 | | -0.02 |
|  |  |  | |  |  | T2 - Windy | | -0.02 | 0.00 | -0.03 | | -0.02 |
| **Average Max Current Speed** | | | | | | | | | |  | |  |
| T1 | 0.14 | 0.01 | | 0.11 | 0.16 | T1 - Wet | | -0.01 | 0.00 | -0.02 | | 0.01 |
| Wet | 0.14 | 0.02 | | 0.11 | 0.17 | T1 - T2 | | 0.01 | 0.01 | -0.01 | | 0.02 |
| T2 | 0.13 | 0.01 | | 0.10 | 0.15 | T1 – Win3dy | | -0.05 | 0.02 | -0.09 | | -0.02 |
| Windy | 0.19 | 0.03 | | 0.14 | 0.24 | Wet - T2 | | 0.01 | 0.01 | 0.00 | | 0.03 |
|  |  |  | |  |  | Wet - Windy | | -0.05 | 0.01 | -0.08 | | -0.01 |
|  |  |  | |  |  | T2 - Windy | | -0.06 | 0.02 | -0.10 | | -0.02 |
| **Average Min Current Speed** | | | | |  |  | |  |  |  | |  |
| T1 | 0.00 | 0.01 | | -0.01 | 0.02 | T1 - Wet | | -0.00 | 0.00 | -0.01 | | -0.00 |
| Wet | 0.01 | 0.01 | | -0.00 | 0.02 | T1 - T2 | | -0.01 | 0.00 | -0.02 | | -0.01 |
| T2 | 0.02 | 0.01 | | 0.01 | 0.03 | T1 - Windy | | -0.03 | 0.00 | -0.03 | | -0.02 |
| Windy | 0.03 | 0.01 | | 0.02 | 0.04 | Wet - T2 | | -0.01 | 0.00 | -0.01 | | -0.01 |
|  |  |  | |  |  | Wet - Windy | | -0.02 | 0.00 | -0.03 | | -0.02 |
|  |  |  | |  |  | T2 - Windy | | -0.01 | 0.00 | -0.02 | | -0.01 |

**Table S13** – GLMM (Gamma – log link) results comparing mean daily current speed (m s ^-1^) between seasons on four nearshore reefs in Kimbe Bay. Pairwise contrasts and 95% confidence intervals are presented based on adjusted Tukeys method. **T1 n = 360 , Wet Season n = 360, T2 n = 368, Windy Season n = 368**

| *Season* | *estimate* | *SE* | *lower.CL* | *upper.CL* | *contrast* | *estimate* | *SE* | | *lower.CL* | *upper.CL* |
| --- | --- | --- | --- | --- | --- | --- | --- | --- | --- | --- |
| **Mean Current Speed** | | | | |  | | |  | |  |
| T1 | 0.057 | 0.006 | 0.046 | 0.068 | T1 - Wet | 0.012 | 0.002 | | 0.008 | 0.016 |
| Wet | 0.045 | 0.006 | 0.034 | 0.056 | T1 - T2 | 0.011 | 0.001 | | 0.007 | 0.015 |
| T2 | 0.046 | 0.006 | 0.035 | 0.057 | T1 - Windy | 0.001 | 0.001 | | -0.003 | 0.005 |
| Windy | 0.056 | 0.006 | 0.045 | 0.067 | Wet - T2 | -0.001 | 0.001 | | -0.005 | 0.003 |
|  |  |  |  |  | Wet - Windy | -0.011 | 0.001 | | -0.015 | -0.007 |
|  |  |  |  |  | T2 - Windy | -0.010 | 0.001 | | -0.014 | -0.006 |
| **Average Max Current Speed** | | | | |  |  |  | |  |  |
| T1 | 0.121 | 0.025 |  |  | T1 - Wet | -0.010 | 0.004 | | -0.020 | 0.001 |
| Wet | 0.130 | 0.025 |  |  | T1 - T2 | 0.005 | 0.004 | | -0.005 | 0.015 |
| T2 | 0.116 | 0.025 |  |  | T1 - Windy | -0.014 | 0.004 | | -0.025 | -0.003 |
| Windy | 0.135 | 0.025 |  |  | Wet - T2 | 0.014 | 0.004 | | 0.005 | 0.024 |
|  |  |  |  |  | Wet - Windy | -0.005 | 0.004 | | -0.014 | 0.005 |
|  |  |  |  |  | T2 - Windy | -0.019 | 0.004 | | -0.029 | -0.009 |
| **Average Min Current Speed** | | | | |  |  |  | |  |  |
| T1 | 0.031 | 0.005 | 0.020 | 0.041 | T1 - Wet | 0.014 | 0.001 | | 0.010 | 0.018 |
| Wet | 0.017 | 0.005 | 0.007 | 0.027 | T1 - T2 | 0.013 | 0.001 | | 0.009 | 0.016 |
| T2 | 0.018 | 0.005 | 0.008 | 0.028 | T1 - Windy | 0.017 | 0.001 | | 0.014 | 0.021 |
| Windy | 0.013 | 0.005 | 0.003 | 0.023 | Wet - T2 | -0.001 | 0.001 | | -0.005 | 0.002 |
|  |  |  |  |  | Wet - Windy | 0.004 | 0.001 | | 0.000 | 0.007 |
|  |  |  |  |  | T2 - Windy | 0.005 | 0.001 | | 0.002 | 0.008 |

**Table S14** – GLMM (gaussian with identity link unless stated) results comparing mean daily temperatures (^o^C) between seasons on four pinnacle reefs in Kimbe Bay. Pairwise contrasts and 95% confidence intervals are presented based on adjusted Tukeys method. T1 n = 360 , Wet Season n = 360, T2 n = 368, Windy Season n = 368

| *Season* | *estimate* | | *SE* | | *lower.CL* | *upper.CL* | | *contrast* | *estimate* | | | *SE* | *lower.CL* | | *upper.CL* | |
| --- | --- | --- | --- | --- | --- | --- | --- | --- | --- | --- | --- | --- | --- | --- | --- | --- |
| **Mean Temperature** | | | |  | |  |  | | | | | |  |  | |  |
| T1 | 29.82 | 0.31 | | 29.21 | | 30.43 | T1 - Wet | | | -0.45 | 0.05 | | -0.58 | -0.32 | |  |
| Wet | 30.26 | 0.31 | | 29.66 | | 30.87 | T1 - T2 | | | -1.30 | 0.05 | | -1.43 | -1.17 | |  |
| T2 | 31.12 | 0.31 | | 30.51 | | 31.72 | T1 - Windy | | | -0.79 | 0.06 | | -0.94 | -0.65 | |  |
| Windy | 30.61 | 0.31 | | 30.00 | | 31.22 | Wet - T2 | | | -0.85 | 0.03 | | -0.94 | -0.77 | |  |
|  |  |  | |  | |  | Wet - Windy | | | -0.35 | 0.04 | | -0.45 | -0.24 | |  |
|  |  |  | |  | |  | T2 - Windy | | | 0.51 | 0.04 | | 0.40 | 0.61 | |  |
| **Average Max Temperature** | | | | | | |  | | |  |  | |  |  | |  |
| T1 | 29.98 | 0.30 | | 29.39 | | 30.57 | T1 - Wet | | | -0.44 | 0.05 | | -0.58 | -0.30 | |  |
| Wet | 30.42 | 0.31 | | 29.82 | | 31.02 | T1 - T2 | | | -1.37 | 0.06 | | -1.52 | -1.22 | |  |
| T2 | 31.35 | 0.33 | | 30.71 | | 31.99 | T1 - Windy | | | -0.80 | 0.06 | | -0.96 | -0.64 | |  |
| Windy | 30.78 | 0.32 | | 30.16 | | 31.40 | Wet - T2 | | | -0.93 | 0.04 | | -1.03 | -0.82 | |  |
|  |  |  | |  | |  | Wet - Windy | | | -0.36 | 0.04 | | -0.47 | -0.24 | |  |
|  |  |  | |  | |  | T2 - Windy | | | 0.57 | 0.05 | | 0.45 | 0.69 | |  |
| **Average Min Temperature** | | | | | |  |  | | |  |  | |  |  | |  |
| T1 | 29.56 | 0.31 | | 28.94 | | 30.17 | T1 - Wet | | | -0.44 | 0.05 | | -0.57 | -0.32 | |  |
| Wet | 30.00 | 0.31 | | 29.39 | | 30.61 | T1 - T2 | | | -1.26 | 0.05 | | -1.38 | -1.13 | |  |
| T2 | 30.81 | 0.31 | | 30.20 | | 31.42 | T1 - Windy | | | -0.81 | 0.06 | | -0.95 | -0.67 | |  |
| Windy | 30.36 | 0.31 | | 29.75 | | 30.98 | Wet - T2 | | | -0.82 | 0.03 | | -0.90 | -0.73 | |  |
|  |  |  | |  | |  | Wet - Windy | | | -0.37 | 0.04 | | -0.47 | -0.27 | |  |
|  |  |  | |  | |  | T2 - Windy | | | 0.45 | 0.04 | | 0.34 | 0.55 | |  |

**Table S15** – GLMM (gaussian with identity link unless stated) results comparing mean daily temperatures (^o^C) between seasons on three offshore reefs in Kimbe Bay. Pairwise contrasts and 95% confidence intervals are presented based on adjusted Tukeys method. T1 n = 276, Wet Season n = 276, T2 n = 276, Windy Season n = 276

| *Season* | *estimate* | *SE* | *lower.CL* | | *upper.CL* | *contrast* | *estimate* | *SE* | *lower.CL* | *upper.CL* |
| --- | --- | --- | --- | --- | --- | --- | --- | --- | --- | --- |
| **Mean Temperature** | | | |  |  |  | | |  |  |
| T1 | 30.34 | 0.34 | 29.68 | | 31.00 | T1 - Wet | -0.88 | 0.05 | -1.01 | -0.76 |
| Wet | 31.22 | 0.34 | 30.56 | | 31.89 | T1 - T2 | -1.05 | 0.05 | -1.19 | -0.91 |
| T2 | 31.39 | 0.34 | 30.73 | | 32.05 | T1 - Windy | -1.85 | 0.06 | -2.00 | -1.71 |
| Windy | 32.19 | 0.34 | 31.53 | | 32.86 | Wet - T2 | -0.17 | 0.05 | -0.29 | -0.05 |
|  |  |  |  | |  | Wet - Windy | -0.97 | 0.05 | -1.10 | -0.84 |
|  |  |  |  | |  | T2 - Windy | -0.80 | 0.05 | -0.94 | -0.66 |
| **Average Max Temperature** | | | |  |  |  |  |  |  |  |
| T1 | 30.48 | 0.35 | 29.80 | | 31.16 | T1 - Wet | -0.91 | 0.05 | -1.04 | -0.78 |
| Wet | 31.39 | 0.34 | 30.71 | | 32.06 | T1 - T2 | -1.11 | 0.06 | -1.26 | -0.97 |
| T2 | 31.60 | 0.35 | 30.92 | | 32.27 | T1 - Windy | -1.90 | 0.06 | -2.05 | -1.74 |
| Windy | 32.38 | 0.35 | 31.70 | | 33.06 | Wet - T2 | -0.21 | 0.05 | -0.33 | -0.08 |
|  |  |  |  | |  | Wet - Windy | -0.99 | 0.05 | -1.13 | -0.86 |
|  |  |  |  | |  | T2 - Windy | -0.78 | 0.06 | -0.93 | -0.64 |
| A**verage Min Temperature** | | | | |  |  |  |  |  |  |
| T1 | 30.11 | 0.34 | 29.45 | | 30.78 | T1 - Wet | -0.88 | 0.05 | -1.00 | -0.75 |
| Wet | 30.99 | 0.34 | 30.32 | | 31.65 | T1 - T2 | -1.02 | 0.05 | -1.16 | -0.88 |
| T2 | 31.14 | 0.34 | 30.47 | | 31.80 | T1 - Windy | -1.80 | 0.06 | -1.95 | -1.65 |
| Windy | 31.91 | 0.34 | 31.25 | | 32.58 | Wet - T2 | -0.15 | 0.05 | -0.27 | -0.03 |
|  |  |  |  | |  | Wet - Windy | -0.92 | 0.05 | -1.06 | -0.79 |
|  |  |  |  | |  | T2 - Windy | -0.78 | 0.05 | -0.92 | -0.64 |

**Table S16** – GLMM (gaussian with identity link unless stated) results comparing mean daily temperatures (^o^C) between seasons on four nearshore reefs in Kimbe Bay. Pairwise contrasts and 95% confidence intervals are presented based on adjusted Tukeys method. T1 n = 360 , Wet Season n = 360, T2 n = 368, Windy Season n = 368

| *Season* | *estimate* | *SE* | *lower.CL* | | *upper.CL* | *contrast* | *estimate* | *SE* | *lower.CL* | | *upper.CL* |
| --- | --- | --- | --- | --- | --- | --- | --- | --- | --- | --- | --- |
| **Mean Temperature** | | | |  |  |  | | | |  |  |
| T1 | 30.11 | 0.40 | 29.33 | | 30.88 | T1 - Wet | -0.51 | 0.06 | -0.67 | | -0.35 |
| Wet | 30.62 | 0.39 | 29.85 | | 31.39 | T1 - T2 | -1.16 | 0.06 | -1.31 | | -1.00 |
| T2 | 31.26 | 0.39 | 30.49 | | 32.03 | T1 - Windy | -0.93 | 0.06 | -1.09 | | -0.76 |
| Windy | 31.03 | 0.39 | 30.26 | | 31.81 | Wet - T2 | -0.64 | 0.05 | -0.78 | | -0.50 |
|  |  |  |  | |  | Wet - Windy | -0.41 | 0.05 | -0.55 | | -0.27 |
|  |  |  |  | |  | T2 - Windy | 0.23 | 0.06 | 0.08 | | 0.37 |
| **Average Max Temperature – Gamma Inverse link** | | | | | |  |  |  |  | |  |
| T1 | 30.22 | 0.38 | 29.47 | | 30.96 | T1 - Wet | -0.55 | 0.06 | -0.71 | | -0.39 |
| Wet | 30.76 | 0.39 | 30.00 | | 31.53 | T1 - T2 | -1.23 | 0.07 | -1.41 | | -1.06 |
| T2 | 31.45 | 0.41 | 30.65 | | 32.25 | T1 - Windy | -0.95 | 0.07 | -1.12 | | -0.77 |
| Windy | 31.16 | 0.40 | 30.37 | | 31.95 | Wet - T2 | -0.68 | 0.06 | -0.83 | | -0.54 |
|  |  |  |  | |  | Wet - Windy | -0.40 | 0.06 | -0.54 | | -0.25 |
|  |  |  |  | |  | T2 - Windy | 0.29 | 0.06 | 0.14 | | 0.44 |
| **Average Min Temperature** | | | | |  |  |  |  |  | |  |
| T1 | 29.89 | 0.39 | 29.12 | | 30.66 | T1 - Wet | -0.50 | 0.06 | -0.66 | | -0.34 |
| Wet | 30.39 | 0.39 | 29.62 | | 31.16 | T1 - T2 | -1.10 | 0.06 | -1.25 | | -0.94 |
| T2 | 30.99 | 0.39 | 30.22 | | 31.75 | T1 - Windy | -0.92 | 0.06 | -1.09 | | -0.76 |
| Windy | 30.81 | 0.39 | 30.04 | | 31.58 | Wet - T2 | -0.60 | 0.05 | -0.74 | | -0.46 |
|  |  |  |  | |  | Wet - Windy | -0.43 | 0.05 | -0.57 | | -0.29 |
|  |  |  |  | |  | T2 - Windy | 0.17 | 0.06 | 0.03 | | 0.32 |

**Table S17 -** Average daily temperature (°C ) and current speed ( m s ^-1^) summaries at each reef type during full duration of deployment September 2018-19. Pinnacles n = (4 reefs), offshore n = (3 reefs) and nearshore n = (4 reefs). N = 6427 logs taken in 10 second intervals at each reef.

| **Site** | **Reeftype** | **Current Speed**  **(m/s)** | **SD** | **Min** | **Max** | **Range** | **Temperature** (^o^C) | **SD** | **Min** | **Max** | **Range** | **Current Direction**  **(Degrees From North)** | **SD** |
| --- | --- | --- | --- | --- | --- | --- | --- | --- | --- | --- | --- | --- | --- |
| Bradford | Pinnacle | 0.0880 | 0.05 | 0.00 | 0.3453 | 0.3453 | 30.97 | 0.53 | 28.75 | 32.84 | 4.09 | 323.47 | 37.25 |
| Inglis | Pinnacle | 0.0852 | 0.05 | 0.0001 | 0.3092 | 0.3091 | 29.70 | 0.51 | 28.14 | 31.35 | 3.21 | 6.63 | 43.40 |
| Joelles | Pinnacle | 0.0852 | 0.04 | 0.0004 | 0.2715 | 0.2711 | 30.20 | 0.58 | 28.66 | 32.01 | 3.35 | 18.05 | 36.29 |
| Kimbe Bommie | Pinnacle | 0.0736 | 0.07 | 0.0004 | 0.6030 | 0.6026 | 30.86 | 0.76 | 29.02 | 32.73 | 3.71 | 358.97 | 34.32 |
| Donna | Nearshore | 0.0572 | 0.04 | 0.0001 | 0.3233 | 0.3232 | 30.65 | 0.48 | 28.75 | 35.25 | 6.50 | 106.13 | 52.00 |
| Lady Di | Nearshore | 0.0389 | 0.03 | 0.0001 | 0.1765 | 0.1764 | 31.09 | 0.49 | 28.66 | 32.12 | 3.36 | 28.69 | 44.97 |
| Madaro | Nearshore | 0.0447 | 0.01 | 0.002 | 0.0913 | 0.0893 | 29.83 | 0.97 | 28.75 | 32.29 | 3.54 | 338.06 | 25.92 |
| Susans | Nearshore | 0.0628 | 0.04 | 0.0001 | 0.4132 | 0.4131 | 31.69 | 1.17 | 25.49 | 31.16 | 5.67 | 68.84 | 57.62 |
| Ottos | Offshore | 0.0419 | 0.04 | 0.0001 | 0.5976 | 0.5975 | 32.02 | 0.85 | 28.93 | 35.24 | 6.31 | 114.42 | 42.65 |
| Ema | Offshore | 0.0412 | 0.04 | 0.0001 | 0.5086 | 0.5085 | 30.81 | 0.47 | 28.84 | 31.92 | 3.08 | 356.47 | 37.10 |
| Hogu | Offshore | 0.0732 | 0.04 | 0.0002 | 0.3318 | 0.3316 | 30.90 | 0.84 | 28.75 | 33.17 | 4.43 | 35.79 | 44.08 |
| Kimbe Island | SST | - | - | - | - | - | 29.05 | 0.57 | 27.48 | 29.80 | 2.32 | - | NA |
| North Ema | SST | - | - | - | - | - | 28.92 | 0.69 | 26.34 | 30.02 | 3.68 | - | NA |
| Ottos | SST | - | - | - | - | - | 29.05 | 0.57 | 27.20 | 29.87 | 2.67 | - | NA |
| Walindi | SST | - | - | - | - | - | 28.80 | 0.84 | 26.06 | 29.90 | 3.84 | - | NA |

**Table S18** – Seasonal ranges of temperature (^o^C) and current speed (m s ^-1^) at pinnacle, nearshore and offshore reefs in Kimbe Bay between September 2018 – September 2019.

|  | **T1** | | | | **Wet Season** | | | | | | | | **T2** | | | | | **Windy Season** | | | | | |
| --- | --- | --- | --- | --- | --- | --- | --- | --- | --- | --- | --- | --- | --- | --- | --- | --- | --- | --- | --- | --- | --- | --- | --- |
|  | **Temperature (^o^C)** | | | | | | | | | | | | | | | | | | | | | | |
| **Reef Type** | **Maximum** | **Minimum** | **Range** |  | | **Maximum** | | **Minimum** | **Range** |  | | **Maximum** | | | **Minimum** | **Range** |  | | | **Maximum** | **Minimum** | **Range** | |
| **Pinnacle** | 30.96 | 28.42 | 2.54 |  | | 31.24 | | 29.07 | 2.17 |  | | 32.46 | | | 29.02 | 3.44 |  | | | 32.55 | 28.84 | 3.71 | |
| **Offshore** | 31.78 | 29.76 | 2.02 |  | | 32.81 | | 30.22 | 2.59 |  | | 33.06 | | | 29.30 | 3.76 |  | | | 34.95 | 30.98 | 3.97 | |
| **Nearshore** | 31.09 | 29.58 | 1.51 |  | | 31.13 | | 30.13 | 1.52 |  | | 34.96 | | | 25.49 | 9.47 |  | | | 33.36 | 29.73 | 2.63 | |
|  | **Current Speed (m s^-1^)** | | | | | | | | | | | | | | | | | | | | | | |
|  | **Maximum** | **Minimum** | **Range** |  | | | **Maximum** | **Minimum** | **Range** |  | **Maximum** | | | **Minimum** | | **Range** |  | | **Maximum** | | **Minimum** | | **Range** |
| **Pinnacle** | 0.2249 | 0.005 | 0.2194 |  | | | 0.3951 | 0.0057 | 0.3951 |  | 0.3148 | | | 0.0058 | | 0.3090 |  | | 0.3148 | | 0.0058 | | 0.3090 |
| **Offshore** | 0.2405 | 0.0020 | 0.2385 |  | | | 0.1840 | 0.0013 | 0.1840 |  | 0.2126 | | | 0.0059 | | 0.2067 |  | | 0.3913 | | 0.0182 | | 0.3731 |
| **Nearshore** | 0.2414 | 0.0012 | 0.2402 |  | | | 0.2257 | 0.0050 | 0.2257 |  | 0.1851 | | | 0.0009 | | 0.1842 |  | | 0.3461 | | 0.0029 | | 0.342 |
|  |  |  |  |  | | |  |  |  |  |  | | |  | |  |  | |  | |  | |  |

**Table S18** - Annual 25^th^, Median, 75^th^ percentiles, Interquartile Ranges (IQR) and Mean Absolute Deviation (MAD) for current speed and temperature at each reef type during 2018-19.

|  | Current Speed (m s -1) | | | | | | Temperature (^o^C) | | | | |
| --- | --- | --- | --- | --- | --- | --- | --- | --- | --- | --- | --- |
| Reef type | 25th | Median | 75th |  | IQR | MAD | 25th | Median | 75th | IQR | MAD |
| Nearshore | 0.040 | 0.048 | 0.061 |  | 0.021 | 0.015 | 30.418 | 30.899 | 31.362 | 0.944 | 0.69 |
| Offshore | 0.033 | 0.048 | 0.071 |  | 0.039 | 0.025 | 30.600 | 31.118 | 32.112 | 1.512 | 1.07 |
| Pinnacle | 0.054 | 0.073 | 0.103 |  | 0.048 | 0.034 | 29.947 | 30.433 | 30.967 | 1.021 | 0.74 |

**SI Table 19** – Permutation test results comparing 25^th^ and 75^th^ percentiles, median and IQR for current speed and temperature between reef types during 2018-19. Tests are based on 5000 permutations at the 95% confidence level.

| Comparison | Current Speed (m s -1) | | | | | | Temperature (^o^C) | | | |
| --- | --- | --- | --- | --- | --- | --- | --- | --- | --- | --- |
| *Adjusted p values* | 25th | Median | 75th |  | IQR | 25th | | Median | 75th | IQR |
| Pinnacle - Offshore | <0.01 | <0.01 | <0.01 |  | <0.01 | <0.01 | | <0.01 | <0.01 | <0.01 |
| Pinnacle - Nearshore | <0.01 | <0.01 | <0.01 |  | <0.01 | <0.01 | | <0.01 | <0.01 | 0.09 |
| Offshore - Nearshore | <0.01 | 0.86 | <0.01 |  | <0.01 | <0.01 | | <0.01 | <0.01 | <0.01 |

**Table S21** Seasonal 25^th^, Median, 75^th^ percentiles, Interquartile Ranges (IQR) and Mean Absolute Deviation (MAD) for current speed and temperature at each reef type during 2018-19.

| Season | Reef Type | Current Speed (m s^-1^) | | | | | Temperature (^o^C) | | | |  |
| --- | --- | --- | --- | --- | --- | --- | --- | --- | --- | --- | --- |
|  |  | 25th | Median | 75th | MAD | IQR | 25th | Median | 75th | IQR | *MAD* |
| T1 | Nearshore | 0.042 | 0.047 | 0.068 | 0.012 | 0.026 | 30.020 | 30.222 | 30.680 | 0.660 | 0.44 |
| T1 | Offshore | 0.029 | 0.039 | 0.054 | 0.017 | 0.025 | 30.164 | 30.453 | 30.783 | 0.619 | 0.47 |
| T1 | Pinnacle | 0.055 | 0.072 | 0.096 | 0.029 | 0.041 | 29.853 | 30.156 | 30.364 | 0.511 | 0.38 |
| T2 | Nearshore | 0.033 | 0.047 | 0.056 | 0.016 | 0.023 | 30.593 | 31.184 | 31.858 | 1.265 | 0.92 |
| T2 | Offshore | 0.030 | 0.048 | 0.084 | 0.033 | 0.054 | 30.707 | 31.381 | 32.429 | 1.722 | 1.38 |
| T2 | Pinnacle | 0.046 | 0.057 | 0.074 | 0.021 | 0.028 | 30.473 | 31.250 | 31.764 | 1.290 | 0.91 |
| Wet | Nearshore | 0.039 | 0.045 | 0.052 | 0.009 | 0.013 | 30.855 | 31.020 | 31.201 | 0.346 | 0.26 |
| Wet | Offshore | 0.032 | 0.046 | 0.061 | 0.021 | 0.028 | 30.863 | 31.076 | 31.966 | 1.102 | 0.46 |
| Wet | Pinnacle | 0.062 | 0.080 | 0.111 | 0.034 | 0.049 | 30.005 | 30.333 | 30.691 | 0.686 | 0.50 |
| Windy | Nearshore | 0.042 | 0.056 | 0.072 | 0.022 | 0.030 | 30.658 | 31.291 | 31.896 | 1.238 | 0.92 |
| Windy | Offshore | 0.050 | 0.087 | 0.096 | 0.027 | 0.047 | 31.839 | 32.242 | 32.540 | 0.701 | 0.51 |
| Windy | Pinnacle | 0.073 | 0.106 | 0.136 | 0.046 | 0.063 | 29.618 | 30.687 | 31.184 | 1.566 | 1.21 |

**Table S21** – Permutation test results comparing 25^th^ and 75^th^ percentiles, median and IQR for current speed and temperature between reef types during each season. Tests are based on 5000 permutations at the 95% confidence level.

| Comparison | | Current Speed (m s^-1^) | | | | | Temperature (^o^C) | | | |
| --- | --- | --- | --- | --- | --- | --- | --- | --- | --- | --- |
|  |  | 25th | Median | 75th | IQR | | 25th | Median | 75th | IQR |
| T1 | Pinnacle – Offshore | <0.01 | <0.01 | <0.01 | <0.01 |  | <0.01 | <0.01 | <0.01 | 0.18 |
| T1 | Pinnacle – Nearshore | <0.01 | <0.01 | <0.01 | <0.01 |  | <0.01 | 0.24 | <0.01 | 0.05 |
| T1 | Offshore - Nearshore | <0.01 | <0.01 | <0.01 | 0.77 |  | <0.01 | <0.01 | <0.01 | 0.42 |
| T2 | Pinnacle – Offshore | <0.01 | <0.01 | <0.01 | <0.01 |  | 0.21 | 0.20 | <0.01 | 0.01 |
| T2 | Pinnacle – Nearshore | <0.01 | <0.01 | <0.01 | 0.09 |  | 0.26 | 0.76 | 0.045 | 0.71 |
| T2 | Offshore - Nearshore | <0.01 | 0.56 | <0.01 | <0.01 |  | 0.25 | 0.20 | <0.01 | 0.01 |
| Wet | Pinnacle – Offshore | 0.24 | <0.01 | 0.08 | <0.01 |  | <0.01 | <0.01 | <0.01 | <0.01 |
| Wet | Pinnacle – Nearshore | <0.01 | <0.01 | <0.01 | <0.01 |  | <0.01 | <0.01 | <0.01 | <0.01 |
| Wet | Offshore - Nearshore | <0.01 | 0.64 | <0.01 | <0.01 |  | 0.63 | 0.10 | <0.01 | <0.01 |
| Windy | Pinnacle – Offshore | <0.01 | <0.01 | <0.01 | 0.05 |  | <0.01 | <0.01 | <0.01 | <0.01 |
| Windy | Pinnacle – Nearshore | <0.01 | <0.01 | <0.01 | <0.01 |  | <0.01 | <0.01 | <0.01 | 0.02 |
| Windy | Offshore - Nearshore | <0.01 | <0.01 | <0.01 | <0.01 |  | <0.01 | <0.01 | <0.01 | <0.01 |

**Table S23** – GLMM (gaussian with identity link) results comparing mean standard deviation in the net heading of current direction between three reef types in Kimbe Bay. Data for the year 2018-19 were used to calculate net heading by vector analysis and standard deviations of these means following Yamartino 1984. Pairwise contrasts and 95% confidence intervals are presented based on adjusted Tukey’s method. Pinnacles n = 4, offshore n = 3, nearshore n = 4.

| *Season* | *estimate* | *SE* | *lower.CL* | *upper.CL* | *contrast* | *estimate* | *SE* | *lower.CL* | *upper.CL* |
| --- | --- | --- | --- | --- | --- | --- | --- | --- | --- |
| **Mean Direction Standard Deviation** | | | |  |  | | |  |  |
| Pinnacle | 37.81 | 4.49 | 27.66 | 47.97 | Pinnacle - Offshore | -3.46 | 6.86 | -22.62 | 15.69 |
| Offshore | 41.28 | 5.19 | 29.54 | 53.01 | Pinnacle - Nearshore | -7.31 | 6.35 | -25.05 | 10.42 |
| Nearshore | 45.13 | 4.49 |  |  | Offshore - Nearshore | -3.85 | 6.86 | -23.01 | 15.30 |

**Table S24** – Site locations (Latitude and Longitude in decimal degrees) and depth of current meter mooring.

| **Site** | **Reeftype** | **Latitude** | **Longitude** | **Current Meter Mooring Depth (m)** |
| --- | --- | --- | --- | --- |
| Bradford Shoals | Offshore Pinnacle | -5.156722222 | 150.2990830 | 23 |
| Inglis Shoals | Offshore Pinnacle | -5.236472222 | 150.1714722 | 21 |
| Joelles | Offshore Pinnacle | -5.199333333 | 150.1916667 | 22 |
| Kimbe Bommie | Offshore Pinnacle | -5.202222222 | 150.3815556 | 25 |
| Donna | Nearshore Emergent | -5.318916667 | 150.1193611 | 21 |
| Lady Di | Nearshore Emergent | -5.442860545 | 150.0984867 | 22 |
| Madaro | Nearshore Emergent | -5.419868834 | 150.0894603 | 23 |
| Susans | Nearshore Emergent | -5.289861111 | 150.1312500 | 21 |
| Ottos | Offshore Emergent | -5.325250000 | 150.2962778 | 24 |
| Ema | Offshore Emergent | -5.193222222 | 150.1565833 | 22 |
| Hogu | Offshore Emergent | -5.334027778 | 150.3907220 | 23 |
